# Age and healthy lifestyle behavior’s disparities and similarities on knowledge of myocardial infarction symptoms and risk factors among public and outpatients in a resource-limited setting, cross-sectional study in greater Gaborone, Botswana

**DOI:** 10.1186/s12872-024-03792-4

**Published:** 2024-03-04

**Authors:** Ookeditse Ookeditse, Kebadiretse K. Ookeditse, Thusego R. Motswakadikgwa, Gosiame Masilo, Yaone Bogatsu, Baleufi C. Lekobe, Mosepele Mosepele, Henrik Schirmer, Stein H. Johnsen

**Affiliations:** 1 Department of Physical Medicine and Rehabilitation, Trust Hospital in Vestfold, Kysthospitalet, Division of Neurorehabilitation Medicine, Tønsberg, Norway; 2 Department of Family Medicine, Larvik County Acute and Emergency Clinic, Larvik, Norway; 3 Faculty of Medicine, University of Botswana, Gaborone, Botswana; 4 Notodden Medical Office, Nottoden, Telemark County, Norway; 5 Department of Internal Medicine, Sidilega Private Hospital, Gaborone, Botswana; 6 Division of Family Medicine, Nanset Medical Clinic, Larvik, Norway; 7 Princess Marina Referral Hospital, Gaborone, Botswana; 8 UIT The Arctic University of Norway, Institute of Clinical Medicine, Tromsø, Norway; 9 Department of Cardiology, Akershus University Hospital, Lørenskog, Norway; 10 Institute of Clinical Medicine, University of Oslo, Oslo, Norway; 11 Department of Neurology, University Hospital of North Norway, Tromsø, Norway

**Keywords:** Myocardial infarction, Heart attack, Symptoms, Risk factors, Outpatients, Public, Awareness, Knowledge, Healthy lifestyle behaviour

## Abstract

**Objectives:**

In this cross-sectional study from Botswana, we investigated knowledge of myocardial infarction (MI) symptoms and risk factors among the general public and outpatients with MI risk factors based on age and lifestyle behaviors, in addition to assessing associations with sociodemographic and MI risk factors.

**Method:**

Open-ended questionnaires about 8 MI symptoms and 10 risk factors, were administered by research assistants to a representative selection of outpatients (*n* = 525) and the public (*n* = 2248). Weight and height were measured in all participants and BMI was calculated. Knowledge scores were compared between the two groups. We examined whether sociodemographic and MI risk factors had impact on the scores. Analyses were further adjusted for lifestyle behavior (smoking status, dietary status and physical activities).

**Results:**

The valid response rate was 97.9% comprising 97.8% for the public (*n* = 2199) and 98.1% for outpatients (*n* = 515). Public respondents (35.2 ± 12.3 years) were younger than outpatients (38.5 ± 12.6 years). The public comprised 56.9% females while outpatients 54.6%.

In general, outpatients had higher knowledge of MI symptoms than the public, with mean scores ± SD of 3.49 ± 2.84 vs 2.80 ± 2.54. Outpatients also had higher knowledge score of MI risk factors than the public, with mean scores, 5.33 ± 3.22 vs 3.82 ± 3.07. For MI symptoms, outpatients were more aware than the public for chest pains among all ages, for arm pain/ numbness, neck/ jaw pain radiating to/ from chest, and feeling sick or looking pallor on the skin among those aged 35–49 years.

Among both the public and outpatients, lower knowledge of both MI symptoms and risk factors was associated with primary education, not residing/working together, history of hypertension, no history of heart disease/stroke, and obesity. There were similarities and disparities on MI knowledge among respondents with various numbers of healthy lifestyle behaviours.

**Conclusion:**

Results call for urgent educational campaigns on awareness and knowledge of MI and using strategies based on age and lifestyle behavior.

**Supplementary Information:**

The online version contains supplementary material available at 10.1186/s12872-024-03792-4.

## Highlights

• This is one of the few studies worldwide assessing awareness and knowledge of MI among outpatients and the public concurrently.

• This is the first study assessing knowledge of MI adjusted for lifestyle behaviors.

• Awareness of MI symptoms might increase awareness of contacting emergency medical services for time-dependent therapy.

• Awareness of MI risk factors might improve prevention.

• Outpatients had higher knowledge than the public for MI symptoms and risk factors.

• There were disparities and similarities of MI knowledge based on number of lifestyle behaviours between outpatients and the public.

• Results call for urgent education and using strategies based on age and lifestyle behaviors.

**Supplementary Information:**

The online version contains supplementary material available at 10.1186/s12872-024-03792-4.

## Introduction

Ischemic heart disease (IHD) was the leading cause of all health loss globally (as well as in each world region) in 2015, with 110.6 million prevalent cases of IHD of which 7.3 million were acute myocardial infarctions (MI, heart attacks) and 8.9 million deaths [1]. The total number of disability-adjusted life-years (DALYs) due to IHD has risen steadily since 1990, reaching 182 million DALYs, 9.14 million deaths in the year 2019, and 197 million prevalent cases of IHD in 2019 [2]. IHD is the leading cause of death in developed countries and is one of the leading causes of disease burden in developing countries [3]. Whereas IHD mortality is decreasing in many developed countries, it is increasing in developing and transitional countries, partly as a result of increasing longevity, urbanization, and lifestyle changes [4]. According to the Global Burden of Diseases 2016 (GBD 2016) study [5–8], IHD was the second leading cause of death in Botswana after HIV/AIDS, with an increment of 9.2% from 2005- 2016.

An acute MI typically manifests as chest pain or feeling of tightness, which may radiate to the arms, neck, jaw, and back, with nausea, vomiting, dyspnoea, and sweating [9]. Uncommonly, in some diabetic or elderly patients, MI may present silently. It may also present with diverse atypical symptoms, for example, with abdominal pain [10]. In all cases, however, the patient’s description of any symptoms is considered alongside electrocardiogram findings and cardiac-specific enzyme assays when deciding on the correct diagnosis and treatment.

Early presentation at a hospital when having acute MI symptoms and improved control of MI risk factors provide greater opportunity for effective acute MI treatment and prevention [11]. Decreasing the time from acute MI onset to hospital presentation and risk reduction depends on the knowledge of acute MI of patients, their family members, and the general population. The awareness of acute MI symptoms and risk factors is essential for the public and outpatients to effectively use thrombolytic therapy and/or percutaneous coronary intervention (PCI) in a timely manner [11, 12]. For example, thrombolysis has the potential to limit myocardial damage and rescue stunned myocardium if administered within 24 h, although it should be administered within 60 min of the onset of pain to confer maximal benefit [11]. Worldwide several community-based studies have been performed to assess public awareness of acute MI risk factors, warning signs, and attitudes intended to take when acute MI is suspected [13–24], but only a few have been conducted in Sub-Saharan Africa, and none in Botswana.

There is a paucity of open-ended studies that have been performed worldwide to assess awareness of MI symptoms [13, 24–28] compared to many closed-ended studies [26, 29–42], while we could not find previous open-ended studies about MI risk factors. Closed-ended studies have shown higher knowledge or awareness of MI compared to open-ended ones since closed-ended questions overestimate the real level of awareness and knowledge, as revealed in previous studies [26, 43]. According to our knowledge, there are also no studies of MI knowledge or awareness based on age in Sub-Saharan Africa or healthy lifestyle behaviors worldwide.

## Objectives:

1. To assess knowledge of myocardial infarction symptoms and risk factors among the public and outpatients based on age and lifestyle behaviors in Botswana.
2. To assess whether respondents’ sociodemographic and myocardial infarction risk factors are associated with this knowledge.

## Methods

### Study design and setting

In this cross-sectional questionnaire study, participants were recruited from greater Gaborone, Botswana. Greater Gaborone is located in the southeastern part of the country. It is the most populated area and comprises six (6) districts (Kgatleng, Kweneng, Southeast, Ngwaketse, Lobatse town and Gaborone city). There were two (2) types of respondents: General public with or without MI risk factors that were recruited from their homes or workplaces in both rural and urban areas. The second type were outpatients who are patients with at least one MI risk factor, recruited from both primary and secondary healthcare facilities while waiting in a queue for or after consultation. Recruiting of respondents from the public and outpatients from healthcare facilities were done concurrently so as to avoid the bias of respondents being interviewed twice. This was clarified by asking the respondents if they have recently participated in a stroke study this year. If the answer was yes, they would not be recruited. For more information about methodology, see eFigure 1a.

Trained research assistants interviewed respondents verbatim. Each interviewer conducted a standardized, structured, one-to-one interview, according to a multi-sectioned questionnaire designed to guide the interview and avoid bias. The interviewer intervened only if asked to clarify any question, without giving correct answers. Respondents were verbally informed about the study and written consent solicited prior to participation.

### Inclusion criteria

For the general public, respondents residing or working in Greater Gaborone, with or without any MI risk factors. No more than two (2) respondents from same family, compound or company were included. Outpatients with at least one MI risk factor visiting healthcare facilities in Greater Gaborone during study period but not admitted. Respondents aged at least 18 years since it is considered adult age. Respondents who understood English or local language, Setswana, and could consent. Only respondents from the randomly selected places and not from the pilot study place were included. Respondents were not allowed to participate in the study more than once.

### Exclusion criteria

Respondents with cognitive/speech difficulties.

### Ethical statement

The study was approved by the Ethics Committee of University of Botswana, Ministry of Health and Wellness in Botswana, Health Research and Development Division (ref. no. HPDME: 13/18/1), and by the Regional Ethics Committee, South East, section C (ref. 2018/1121/REK sør-øst A), Norway. The study was reported in accordance with the Strengthening the Reporting of Observational Studies in cross sectional survey (STROBE) reporting guideline [44].

### Data collection instrument

The survey instruments were adapted from previous surveys [13, 24, 25, 27, 28] with some modifications to reflect information based on Centers for Diseases Control and Prevention (CDC), World Health Organization (WHO), and European Society of Cardiology guidelines [45–47]. We tested the questionnaire in a pilot study with a sample of thirty-six respondents and some changes were made in the wording of questions based on the result of the pilot study. The questionnaire instruments were anonymous, electronic-based using tablets (Samsung Galaxy Tab 3 Lite, 7.0 Android, South Korea), standardized, written, and administered in English or local language (Setswana), open-ended in nature, and categorized into 4 sections (eFigure 1b). Assessment of MI symptoms and risk factors was performed through open-ended questions. Section 1 included sociodemographic factors, Sect. 2 about awareness of MI symptoms, Sect. 3 about awareness of MI risk factors, and Sect. 4 about self-reported and calculated MI risk factors (Body mass index (BMI)).

For Sects. 2 and 3, each correct answer scored 1 point and was considered being aware. Each incorrect, unanswered, or unknown answer scored 0 point and was considered unaware.

#### Sociodemographic characteristics

Variables included in this study were age (18–34 years, 35–49 years and ≥ 50 years), gender (male and female), education level (none/ unknown/ primary, secondary and tertiary), medical insurance (yes and no), and residing/ working together status (yes and no).

#### Awareness and knowledge of MI symptoms

Awareness of eight MI symptoms was assessed by individual’s response to an open-ended question (eFigure 1b): “Can you name symptoms of heart attack?” Awareness was defined as awareness rate of each recalled symptom, while knowledge was measured as mean scores of total recalled symptoms out of eight.

#### Awareness and knowledge of MI risk factors

Awareness of ten MI risk factors was assessed by individual’s responses to open-ended questions (eFigure 1b): “Can you name conditions or lifestyle habits that may predispose to heart attack?” Each correct answer scored 1 point and was considered being aware. Each incorrect, unanswered, or unknown answer scored 0 point and was considered unaware. Knowledge was measured as mean scores of total recalled symptoms out of ten.

#### Respondents’ heart attack risk factors

Included hypertension, grouped 4 cardiovascular risk factors (CVDS: diabetes, dyslipidemia, prior stroke, or heart diseases because of low numbers of these respondents), family history of stroke, heart diseases or both (at least in one family member in the first generation), history of Human immunodeficiency virus (HIV/AIDS): (yes or no), psychiatric diseases (depression/ anxiety): (yes or no), smoking (non-smokers, former, or current): Current smokers were individuals who smoked at least 1 tobacco product daily in the previous 12 months, including those who had quit within the past year. Former smokers had quit more than 1 year earlier, while non-smokers had never used tobacco products. Alcohol drinking (non-drinkers, former, or current): Current drinkers were individuals who drank alcohol regularly in the previous 12 months, including those who had quit within the past year. Former drinkers had quit more than 1 year earlier, while non-drinkers had never used alcohol. Perceived dietary status (healthy or unhealthy).

Participants were asked if they perceived their weight as underweight, normal, overweight, or obese. Body weight was measured in kilograms (to the second decimal place) by a self-zeroing digital weight scale for adults dressed in light clothes without shoes. Safeway self-zeroing digital weight scales (Safeway Scale, South Africa) were used after calibration. Height was measured twice to the nearest millimeter using a fixed plastic, non-elastic stadiometer, and average height calculated. BMI was calculated, and classified as defined by the WHO and National Institutes of Health (NIH) i.e., underweight as BMI < 18.5 kg/m^2^, normal BMI 18.5- < 25 kg/m^2^, overweight 25- < 30 kg/m^2^, and obesity as ≥ 30 kg/m^2^ [48, 49]. Perceived physical activity intensities were also recorded (none, light, moderate or high physical intensity).

No smoking status, healthy dietary status and physical activities were further categorized into 3 groups depending on the number of healthy lifestyle behaviors i.e., 0 for none healthy lifestyle (LS0), 1 for 1 lifestyle behavior (LS1), and ≥ 2 for ≥ 2 lifestyle behaviors (LS2).

### Statistical analysis

Continuous variables were expressed as means ± standard deviation (SD). Categorical and ordinal variables were expressed as frequency (n) and proportion (%) of the overall sample or subgroups. Outpatients and public groups’ awareness of MI symptoms and risk factors were compared using chi-squared after stratifying for age because of significant age differences between them. Comparison of acknowledging own MI risk factors was also done by using chi-square test in addition to odds ratio.

One-way ANOVA or independent-samples t-test analysis was used to assess the association of respondents’ sociodemographic and MI risk factors with knowledge of MI symptoms and risk factors if the data was normally distributed, otherwise non-parametric analysis would be used. Two-way ANOVA was used to assess healthy lifestyle behaviors adjusted sociodemographic and MI risk factors’ association with MI knowledge scores. Bonferroni analysis was used to correct for multiple comparisons. Statistical tests were two-tailed and reported statistically significant at *p* < 0.05. All statistical analyses were completed using SPSS 27 statistical software (SPSS Inc., Chicago, Illinois, USA).

## Results

Between June-October 2019, we interviewed 2773 respondents. We excluded 59 participants because either they did not want to participate, had missing consent or substantial information necessary for the study (eFigure 2). We had a valid response of 2714 respondents (97.9%), comprising 2199 of the public (97.8%) and 515 of the outpatients (98.1%). Public respondents were younger than outpatients (35.2 ± 12.3 years, age range 18–82 years vs 38.5 ± 12.6 years, age range 18–78 years). Public respondents comprised 56.9% females while outpatients 54.6% females. More information on respondents’ characteristics is shown in Table 1.

**Table 1:** Sociodemographic and myocardial infarction risk factors among respondents

	Total *n*=2714 n (%)	Public *n*=2199 n (%)	Outpatients *n*=515 n (%)	*p*
**Sociodemographic factors**				
**Gender**				
Female	1532(56.4)	1251(56.9)	281(54.6)	0.338
Male	1182(43.6)	948(43.1)	234(45.4)	
** Age category (years)**				
18-34	1447(53.3)	1238(56.3)	209(40.6)	<0.001
35-49	894(32.9)	683(31.1)	211(41.0)	
>50	373(13.7)	278(12.6)	95(18.4)	
** Education level**				
Primary, or unknown	307(11.3)	255(11.6)	52(10.1)	0.486
Secondary	1311(48.3)	1052(47.8)	259(50.3)	
Tertiary	1096(40.4)	892(40.6)	204(39.6)	
** Medical insurance**				
Yes	373(13.7)	274(12.5)	99(19.2)	<0.001
No, unknown	2341(86.3)	1925(87.5)	416(80.8)	
** Residing/working together**				
Yes	857(31.6)	673(30.6)	184(35.7)	0.024
No	1857(68.4)	1526(69.4)	331(64.3)	
**Self-reported risk factors**				
**History of hypertension**				
Yes	258(9.5)	161(7.3)	97(18.8)	<0.001
No, unknown	2456(90.5)	2038(92.7)	418(81.2)	
**History of CVDS**				
Yes	131(4.8)	70(3.2)	61(11.8)	<0.001
No	2583(95.2)	2129(96.8)	454(88.2)	
**Family history of stroke/heart diseases**				
None	760(28.0)	711(32.3)	49(9.5)	<0.001
Both heart diseases and stroke	739(27.2)	566(25.7)	173(33.6)	
Heart diseases	851(31.4)	649(29.5)	202(39.2)	
Stroke	364(13.4)	273(12.4)	91(17.7)	
**BMI**				
Underweight	95(3.5)	78(3.5)	17(3.3)	0.006
Normal, unknown	2048(75.5)	1651(75.1)	397(77.1)	
Overweight	539(19.9)	451(20.5)	88(17.1)	
Obesity	32(1.2)	19(0.9)	13(2.5)	
**Healthy dietary status**				
No, unknown	1039(38.3)	909(41.3)	130(25.2)	<0.001
Yes	1675(61.7)	1290(58.7)	385(74.8)	
**Alcohol consumption**				
No, unknown	1907(70.3)	1559(70.9)	348(67.6)	0.003
Current	730(26.9)	589(26.8)	141(27.4)	
Former	77(2.8)	51(2.3)	26(5.0)	
**Smoking status**				
No, unknown	2297(84.6)	1888(85.9)	409(79.4)	<0.001
Current	355(13.1)	271(12.3)	84(16.3)	
Former	62(2.3)	40(1.8)	22(4.3)	
**Intensity of physical activity**				
None, unknown	1860(68.5)	1534(69.8)	326(63.3)	0.004
Light	225(8.3)	174(7.9)	51(9.9)	
Moderate	507(18.7)	387(17.6)	120(23.3)	
High	122(4.5)	104(4.7)	18(3.5)	
**History of HIV/AIDS **				
Yes	491(18.1)	277(12.6)	214(41.6)	<0.001
No , unknown	2223(81.9)	1922(87.4)	301(58.4)	
**History of psychiatric diseases**				
Yes	90(3.3)	0(0)	90(17.5)	<0.001
No	2624(96.7)	2199(100.0)	425(82.5)	
**Calculated risk factors**				
**BMI**				
Underweight (<18.5)	99(3.6)	87(4.0)	12(2.3)	0.001
Normal (18.5<25), unknown	1377(50.7)	1079(49.1)	298(57.9)	
Overweight (25<30)	742(27.3)	628(28.6)	114(22.1)	
Obesity (>30)	496(18.3)	405(18.4)	91(17.7)	

*NA* not applicable, *CVDS* cardiovascular risk factors (diabetes, dyslipidemia, stroke, or heart diseases)

Psychiatric diseases: depression or anxiety, *BMI* Body Mass Index

### Awareness of MI symptoms

Among all age-group, outpatients were more aware than the public of central chest pain due to differences among all individual age groups (Table 2). Outpatients were also more aware than the public of arm pain/numbness and neck/jaw pain radiating to from chest due to difference among those aged 35–49 years. Outpatients aged 35–49 years were more aware than the public of feeling sick or looking pallor on the skin. Outpatients aged 18–34 years had the highest rate for central chest pain while the public aged 35–49 years had shortness of breath. Both the public and outpatients aged ≥ 50 years had the lowest awareness for nausea (6.5% and 7.4%) respectively).

**Table 2:** Awareness of acute myocardial infarction symptoms between public and outpatients stratified by age

Age (years)	Public		Outpatients		
	n	Aware (%)	n	Aware (%)	p*
**Shortness of breath**					
All	2199	1439(65.4)	515	320(62.1)	0.550
18-34	1238	826(66.7)	209	119(56.9)	0.239
35-49	683	476(69.7)	211	136(64.5)	0.568
>50	278	137(49.3)	95	65(68.4)	0.135
**Central chest pain**					
All	2199	921(41.9)	515	349(67.8)	<0.001
18-34	1238	578(46.7)	209	157(75.1)	<0.001
35-49	683	264(38.7)	211	136(64.5)	0.001
>50	278	79(28.4)	95	56(58.9)	0.005
**Fainting/ dizziness**					
All	2199	1191(54.2)	515	310(60.2)	0.249
18-34	1238	691(55.8)	209	117(56.0)	0.983
35-49	683	384(56.2)	211	131(62.1)	0.491
>50	278	116(41.7)	95	62(65.3)	0.054
**Arm pain/ numbness**					
All	2199	555(25.2)	515	197(38.2)	<0.001
18-34	1238	309(25.0)	209	65(31.1)	0.271
35-49	683	186(27.2)	211	96(45.5)	0.006
>50	278	60(21.6)	95	36(37.9)	0.074
**Feeling sick or looking pallor on the skin**					
All	2199	640(29.1)	515	184(35.7)	0.093
18-34	1238	388(31.3)	209	65(31.1)	0.970
35-49	683	192(28.1)	211	89(42.2)	0.032
>50	278	60(21.6)	95	30(31.6)	0.245
**Sweating and clammy skin**					
All	2199	627(28.5)	515	171(33.2)	0.222
18-34	1238	368(29.7)	209	56(26.8)	0.605
35-49	683	203(29.7)	211	87(41.2)	0.081
>50	278	56(20.1)	95	28(29.5)	0.264
**Neck/ jaw pain radiating from chest**					
All	2199	569(25.9)	515	187(36.3)	0.007
18-34	1238	349(28.2)	209	72(34.4)	0.290
35-49	683	169(24.7)	211	87(41.2)	0.009
>50	278	51(18.3)	95	28(29.5)	0.172
**Nausea**					
All	2199	224(10.2)	515	77(15.0)	0.051
18-34	1238	134(10.8)	209	32(15.3)	0.241
35-49	683	72(10.5)	211	38(18.0)	0.075
>50	278	18(6.5)	95	7(7.4)	0.848

p*: calculated using chi-squared

### Awareness of MI risk factors

Among all age-group, outpatients were more aware than the public of smoking and diabetes as risk factors due to differences in all age groups (Table 3). Also, outpatients were more aware than the public of hypertension and dyslipidemia due to differences among age groups 18–34 and 35–49, while of heavy alcohol intake, previous stroke, heart diseases, and family history of stroke/ heart diseases due to differences among age groups 34–49 and ≥ 50. Highest awareness was present for obesity among outpatients aged 18–34 years (85.2%), while lowest awareness was for family history of stroke/ heart diseases (10.1%) among public aged ≥ 50 years.

**Table 3:** Awareness of myocardial infarction risk factors between public and outpatients stratified by age

Age (years)	Public		Outpatients		
	n	Aware (%)	n	Aware (%)	p*
**Hypertension**					
All	2199	1291(58.7)	515	411(79.8)	<0.001
18-34	1238	719(58.1)	209	165(78.9)	0.017
35-49	683	423(61.9)	211	171(81.0)	0.042
>50	278	149(53.6)	95	75(78.9)	0.064
**Dyslipidemia**					
All	2199	520(23.6)	515	209(40.6)	<0.001
18-34	1238	295(23.8)	209	75(35.9)	0.036
35-49	683	173(25.3)	211	101(47.9)	<0.001
>50	278	52(18.7)	95	33(34.7)	0.061
**Diabetes**					
All	2199	779(35.4)	515	291(56.5)	<0.001
18-34	1238	439(35.5)	209	111(53.1)	0.012
35-49	683	258(37.8)	211	121(57.3)	0.011
>50	278	82(29.5)	95	59(62.1)	0.004
**Sedentary lifestyle**					
All	2199	884(40.2)	515	243(47.2)	0.127
18-34	1238	510(41.2)	209	88(42.1)	0.896
35-49	683	296(43.3)	211	112(53.1)	0.207
>50	278	78(28.1)	95	43(45.3)	0.088
**Heavy alcohol intake**					
All	2199	781(35.5)	515	264(51.3)	<0.001
18-34	1238	457(36.9)	209	90(43.1)	0.357
35-49	683	252(36.9)	211	123(58.3)	0.005
>50	278	72(25.9)	95	51(53.7)	0.008
**Previous stroke**					
All	2199	551(25.1)	515	193(37.5)	0.002
18-34	1238	326(26.3)	209	62(29.7)	0.549
35-49	683	180(26.4)	211	96(45.5)	0.004
>50	278	45(16.2)	95	35(36.8)	0.015
**Heart diseases**					
All	2199	879(40.0)	515	243(47.2)	0.114
18-34	1238	531(42.9)	209	79(37.8)	0.449
35-49	683	273(40.0)	211	112(53.1)	0.084
>50	278	75(27.0)	95	52(54.7)	0.009
**Family history of stroke/ heart diseases**					
All	2199	321(14.6)	515	137(26.6)	<0.001
18-34	1238	193(15.6)	209	42(20.1)	0.310
35-49	683	100(14.6)	211	72(34.1)	<0.001
>50	278	28(10.1)	95	23(24.2)	0.038
**Obesity**					
All	2199	1471(66.9)	515	400(77.7)	0.067
18-34	1238	848(68.5)	209	178(85.2)	0.072
35-49	683	475(69.5)	211	155(73.5)	0.678
>50	278	148(53.2)	95	67(70.5)	0.192
**Smoking**					
All	2199	927(42.2)	515	352(68.3)	<0.001
18-34	1238	525(42.4)	209	131(62.7)	0.008
35-49	683	312(45.7)	211	152(72.0)	0.002
>50	278	90(32.4)	95	69(72.6)	<0.001

p*: calculated using chi-squared

### Acknowledging of own MI risk factors

In general, outpatients with the following risk factors were more aware than the public in recognizing them: family history of heart diseases/ stroke (26.8% vs 17.7%) due to group differences among those aged > 34 years, smoking (66.7% vs 31.7%) due to group differences among those aged < 35 years and > 49 years, and sedentary lifestyle (51.8% vs 37.5%) due to group differences among those aged < 35 years (eTable 1).

Odds of having family history of heart diseases/ stroke while acknowledging it as a risk factor were 2.4 times higher than not having it but acknowledging it due to all ages. For being overweight and acknowledging it, odds were 1.2 times higher than normal weight and acknowledging it due to those aged ≥ 50 years. However, for smoking, sedentary lifestyle, and being obese, odds for acknowledging them as risk factors were 0.7- 0.8 times lower than not having them but acknowledging them due to those 18–34 years old, older than 34 years, and ≥ 50 years old respectively (eTable 2). Almost similar trends were observed among outpatients and the public.

### Knowledge scores of MI symptoms and risk factors

**Fig.1:**
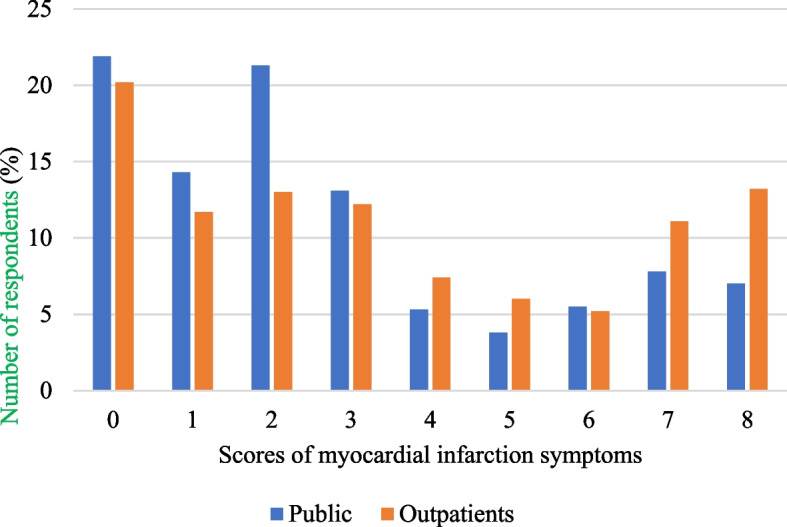
Scores of myocardial infarction symptoms among respondents

**Fig. 2:**
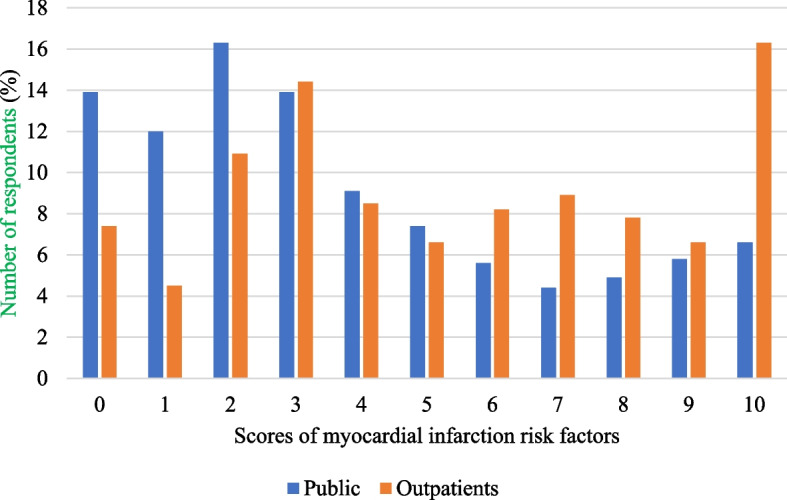
Scores of myocardial infarction risk factors among respondents

For MI symptoms, 13.2% of outpatients vs 7.0% of public spontaneously recalled all 8 symptoms while 79.8% of outpatients vs 78.1% of the public recalled at least one symptom (Fig. 1). Mean knowledge score was 3.49 ± 2.84 for outpatients and 2.80 ± 2.54 for the public (eTable 3). For MI risk factors, 16.3% of outpatients vs 6.6% of the public spontaneously recalled all 10 risk factors while 92.6% of outpatients vs 86.1% of the public recalled at least one risk factor (Fig. 2). Mean knowledge score was 5.33 ± 3.22 for outpatients and 3.82 ± 3.07 for the public.

### Sociodemographic factors’ association with MI knowledge scores

Among both the public and outpatients, lower knowledge of both MI symptoms and risk factors was associated with primary education, not residing/working together, history of hypertension, no history of heart disease/stroke, and obesity (eTable 3). Among both the public and outpatients, lower knowledge of MI risk factors was associated with no medical insurance and no history for HIV/AIDS.

Among the public, lower knowledge of both MI symptoms and risk factors was associated with age ≥ 50 years, current smokers, current drinkers, and no physical activity, while among outpatients, it was associated with secondary education, history of heart disease, and history of psychiatric diseases. Among public, lower knowledge of MI risk factors was associated with no healthy diet, while among outpatients, it was associated with not being married/cohabiting. Among outpatients, lower knowledge of MI symptoms was associated with no medical insurance, no healthy diet, history of CVDS, no healthy diet, and no history of HIV/AIDS.

### Healthy lifestyle behaviors’ association with MI knowledge scores

For those who spontaneously recalled all 8 MI symptoms among those with LS0 were 21.6% of outpatients vs 8.2% of the public while for those with LS1, 12.6% of outpatients vs 6.5% of the public, and for those with LS2, 10.8% of outpatients vs 7.0% of the public (eFigure 3a and eFigure 3b). Mean knowledge scores for those with LS0 was 3.64 ± 3.39 for outpatients vs 2.71 ± 2.67 for public, while for those with LS1 was 3.57 ± 2.77 for outpatients vs 2.70 ± 2.49 for public, and for those with LS2 was 3.32 ± 2.69 for outpatients vs 3.09 ± 2.48 for public. For those who spontaneously recalled all 10 MI risk factors among those with LS0 were 18.9% of outpatients vs 8.7% of the public while for those with LS1, 14.6% of outpatients vs 5.0% of the public, and for those with LS2, 17.5% of outpatients vs 7.5% of public (eFigure 4a and eFigure 4b). Mean knowledge scores for those with LS0 was 5.49 ± 3.52 for outpatients vs 3.53 ± 3.29 for public, while for those with LS1 was 5.51 ± 3.11 for outpatients vs 3.76 ± 2.93 for public, and for those with LS2 was 5.03 ± 3.25 for outpatients vs 4.23 ± 3.04 for public.

Among both public and outpatients with LS0, lower knowledge of both MI symptoms and risk factors was associated with not residing/working together (eTable 4). Among both public and outpatients with LS0, lower knowledge of MI symptoms was associated with history of hypertension. Among both public and outpatients with LS0, lower knowledge of MI risk factors was associated with age ≥ 50 years, primary education, and no family history of heart disease/stroke. Among public with LS0, lower knowledge of both MI symptoms and risk factors was associated with secondary education, while among outpatients with LS0, it was associated with history of CVDS, and obesity. Among public with LS0, lower knowledge of MI symptoms was associated with age ≥ 50 years, primary education, medical insurance, and no family history of heart diseases/stroke. Among public with LS0, lower knowledge of MI risk factors was associated with age 35–49 years, current drinkers, and no history of HIV/AIDS while among outpatients with LS0, it was associated with history of hypertension. Among respondents with LS1, refer to eTable 4.

Among both public and outpatients with LS2, lower knowledge of MI risk factors was associated with no history of HIV/AIDS, no family history of heart disease/stroke, and family history of heart disease. Among the public with LS2, lower knowledge of both MI symptoms and risk factors was associated with primary education, and current drinkers while among outpatients with LS2, it was associated with no medical insurance, and history of psychiatric diseases. Among public with LS2, lower knowledge MI symptoms was associated with not residing/working together, not married/cohabiting, no family history of heart diseases/stroke, family history of heart diseases/stroke, family history of heart diseases. This contrasts outpatients with LS2, where lower knowledge was associated with history of hypertension, and no history of HIV/AIDS. Among public with LS2, lower knowledge of MI risk factors was associated with age 18–34 years while among outpatients with LS2, it was associated with not married/cohabiting, residing together, and non-drinkers.

## Discussion

Our study demonstrated that for MI symptoms, outpatients were more aware than the public for chest pains among all ages, for arm pain/ numbness, neck/ jaw pain radiating to/ from chest, and feeling sick or looking pallor on the skin among those aged 35–49 years. For MI risk factors, outpatients were more aware than the public for smoking and diabetes among all ages, for hypertension and dyslipidaemia among ages 18–34 and 35–49 years, for heavy alcohol intake, previous stroke, history of heart diseases and family history of stroke/ heart diseases among ages 35–49 and ≥ 50 years. Among outpatients, the most frequently recalled risk factors in all age-groups (79–81%) were hypertension and obesity among those aged 18–34 years (85%) while also similar findings were observed among but aged 18–49 years. Outpatients had higher awareness than the public for seven out of ten MI risk factors. Exceptions were for obesity and smoking. Despite this level of awareness, outpatients more often recalled their own MI risk factors than the public, especially among those with a family history of heart diseases/ stroke, smokers, and those with a sedentary lifestyle. This could be explained by outpatients being more frequently in contact with healthcare professionals, being given more specific information, and may have more interest in learning about their diseases. Among those aged less than 50 years, it might be because they have other different sources of information like social media, television and magazines among others which the older ones do not have access to, they probably understand better the way healthcare professionals explain or relay information, in addition to that MI information might have been taught or emphasized at schools the last few years since it is a global burden.

There were similarities and disparities on MI knowledge among respondents with various numbers of healthy lifestyle behaviours. For those with non-healthy lifestyle among both public and outpatients, lower knowledge of both MI symptoms and risk factors was associated with not residing/working together, while those with LS1 had in addition primary education, history of hypertension, and obesity. This could be due to that those with hypertension and obesity are unaware of these as risk factors because they have low education level. Residing/working together seems to be helping in having better knowledge may be due to that people discuss important healthy issues with their colleagues at work and home. For risk factors among those with LS2, lower knowledge was associated with no history of HIV/AIDS, no family history of heart disease/stroke, and family history of heart disease. Among public with LS0, lower knowledge of both MI symptoms and risk factors was associated with secondary education, while among outpatients with LS0, it was associated with history of CVDS, and obesity. Among public with LS1, lower knowledge of both MI symptoms and risk factors was associated with age ≥ 50 years while among outpatients with LS1, it was associated with secondary education, no medical insurance, and history of psychiatric diseases. It is a concern that history of psychiatric diseases, no medical insurance, no history of HIV/AIDS, obesity, history of CVDS, history of hypertension, family history of heart diseases, high age, primary to secondary education, and current drinkers among various lifestyle behaviours are factors that were associated with lower knowledge. Therefore, these subgroups need to be targeted if we are to reduce disability and mortality related to MI in Sub-Saharan Africa. Our study demonstrated that having LS2 with lower MI knowledge was not associated with any specific/less number of MI risk factors.

In our study, the most frequently recalled symptoms by outpatients were central chest pain among those aged 18–34 years (75%), shortness of breath among those aged ≥ 50 years (68%), followed by central chest pain, shortness of breath among 35–49-year-olds and fainting/ dizziness among ≥ 50-year-olds (65% each). It resonates with some studies [27, 28] that had central chest pain (82–92%) and shortness of breath (39%) as the most frequently recalled symptoms even though they were not age stratified. It resonates partly with one study [22] that demonstrated shortness of breath as the most frequently recalled symptom among those aged 65-years while ours also among those aged ≥ 50 years. This study also contradicts ours that demonstrated central chest pain as the most frequently recalled symptom among those < 50 years old.

Among the public, the most frequently recalled symptoms were shortness of breath among those aged 18–34 years and 35–49 years (67–70%), and fainting/ dizziness among those aged 18–34 years and 35–49 years (56%). This is in line with some studies showing shortness of breath [13, 24, 25, 50] as the most frequently recalled symptoms. Some other studies, however, found that central chest pain [13, 24, 25, 50] and arm pain/ numbness [13, 24] was most frequently recalled, while fainting/ dizziness was one of the lowest [30]. The differences in these studies could be attributed to no age-stratification and differences in the study populations.

Outpatients had higher knowledge score of MI symptoms than the public (3.49 vs. 2.80). Outpatients had higher knowledge of MI risk factors than the public (5.33 vs. 3.82)*.* Based on number of lifestyle behaviours, at least 15% of outpatients spontaneously recalled all 10 MI risk factors compared to less than 10% of the public. Similar findings were also observed for MI symptoms, with at least 11% of outpatients vs ≤ 8% of the public spontaneously recalling all 8 symptoms. This could be due to outpatients’ own experience and healthcare professionals’ emphasis on the relevant MI symptoms and risk factors. Outpatients may also have more interest and motivation in learning about their diseases and risk factors, compared to the public.

Our study demonstrated an association of higher knowledge of MI symptoms with age 18–49 years among the public. Our study did not show any association with gender. This resonates with one study that did not show any association with gender [24] and partly with another study that showed an association with middle-aged persons [13]. It contrasts with other studies that did not show any association with age [24, 25]. We found that lower knowledge of MI symptoms was associated with low education level among both public and outpatients, and also among those with one lifestyle behavior. A study from Tanzania found no such association [25]. On the other hand, our study demonstrated association of lower knowledge of MI symptoms with a history of CVDS among outpatients in general and those outpatients with non-healthy lifestyle behavior. This contrasts some studies that demonstrated higher knowledge among respondents with previous CVDS (heart attack) [13, 24, 27]. In our study, lower knowledge of MI symptoms was associated with no family history of stroke/ heart diseases among the public with none or one healthy lifestyle behavior. This contrasts one study that demonstrated higher knowledge with respondents who had relatives with heart attack [24]. Our study demonstrated that lower knowledge was associated with history of hypertension for both outpatients and the public with LS0-1, in addition to outpatients with also LS2. We showed also lower knowledge with obesity among both outpatients and the public. Despite that for dichotomous subgroups, differences were also contributed by large effect sizes, results still demonstrate that there is still lower knowledge among respondents with MI risk factors and that more education campaigns are still needed. Most of the variations with previous studies could be attributed to differences in the study population, how age was defined and if they were adjusted for healthy lifestyle behavior or not, year of research study and place.

There were no previous open-ended studies on MI risk factors found for comparison with our study. Some of the differences in knowledge of MI risk factors were also due to large effect sizes among dichotomous subgroups. Despite this, several studies have demonstrated gender differences in the pathophysiology of atherosclerosis, cardiovascular risk factors, diagnosis of coronary artery diseases and valvular heart disease, and management and outcomes after acute coronary syndromes and valvular repair [51], while we did not show any gender disparities in knowledge of MI risk factors.

### Strength and limitations

Information was obtained by an open-ended questionnaire among public and outpatients concurrently. A very high response was attained and therefore these results represent best current knowledge of the public and outpatients in greater Gaborone. All information from the questionnaires was collected through standardized face-to-face interviews. We compared our results with mostly previous open-ended studies for the public and patients.

There are, however, limitations to this study. Not all MI symptoms and risk factors included in this study should be weighted equally because some are easily identifiable and more common than others. Some of the subgroups had small samples, reducing the statistical power to calculate differences. Other studies have considered knowledge or awareness differently while we resorted to lowest or highest mean score. Self-reported factors/characteristics are prone to bias. Lastly, there may be differences in demographic factors between responders and non-responders that we are unable to account for.

## Conclusion

Despite outpatients demonstrating higher awareness and knowledge of MI symptoms and risk factors than the public, awareness and knowledge were poor to suboptimal. Our study calls for more and strategized education campaigns targeting the population based on age and healthy lifestyle behaviors. Such campaigns must focus on all aspects of MI, including prevention strategies and lifestyle intervention as well as early recognition of MI symptoms and contact with emergency medical services (EMS).

### Supplementary Information

**Supplementary Material 1. **

**Supplementary Material 2. **

**Supplementary Material 3. **

**Supplementary Material 4. **

**Supplementary Material 5. **

**Supplementary Material 6. **

**Supplementary Material 7. **

**Supplementary Material 8. **

## Data Availability

The datasets used and analyzed during the current study are available from the main author on a reasonable request.
