# Supplementary material for: Age and healthy lifestyle behavior’s disparities and similarities on knowledge of myocardial infarction symptoms and risk factors among public and outpatients in a resource-limited setting, cross-sectional study in greater Gaborone, Botswana
Source: BMC Cardiovasc Disord. 2024 Mar 4;24:140. doi: 10.1186/s12872-024-03792-4 (PMC10910839; doi:10.1186/s12872-024-03792-4)
Supplement: Supplementary file 1 — Supplementary Material 1. [file 12872_2024_3792_MOESM1_ESM.docx]

**eFigure 1a. Study Methods**

**Sampling of study places and population**

All names of the four (4) districts in Greater Gaborone except Gaborone City and Lobatse town were put in a box, and two (2) names were blindly selected from the box as study places in addition to Gaborone city and Lobatse town. This applied also to healthcare facilities and communities in the selected districts.

For Gaborone city, all areas were categorized into three socio-economic groups i.e., low, middle and high income. Names of more than three (3) areas with similar socio-economic category were put in a box and three (3) names were blindly selected to represent that specific category. If they were only two areas in the same category, they were both included. This included Phakalane and Extension (high income), Blocks and Broadhurst (middle income), Old Naledi, Bontleng, and Tsolamosese (low income).

Respondents from the general public were recruited from their homes or workplaces in both rural and urban areas. Rural areas included Molepolole in the Kweneng, Mathubudukwane and Mochudi in Kgatleng, while urban areas included the capital city, Gaborone. For the general public in each selected area, we selected households or companies with odd numbers. No more than two respondents from the same family/compound/ company were interviewed.

For outpatients, they were interviewed while waiting in a queue for or after consultation, and only odd numbers were interviewed. Outpatients were recruited from both primary and secondary healthcare facilities. They were screened by a healthcare facility nurse/ doctor for eligibility before informed consent was given. Primary healthcare refers to first line of healthcare, included medical health clinics for outpatients where there are nurses and/ or medical doctors which can refer patients to district hospitals. Public health clinics in all districts are run by District Health Management Team (DHMT). Primary healthcare included two out of six DHMTs in greater Gaborone (Nkoyaphiri clinic and Phuthadikobo clinic in Kweneng, and Phaphane clinic and Mathubudukwane clinic in Kgatleng). Secondary healthcare refers to where primary healthcare refers to, consists of hospital healthcare staff (general doctors and some specialists (internal medicine, general surgery), nurses, laboratory technicians, etc.), and is capable of admitting patients. Secondary healthcare included district hospitals (Scottish Livingstone hospital in Kweneng, and Deborah Retief Memorial Hospital in Kgatleng), and the only tertiary referral psychiatric hospital in the country (Sbrana Psychiatric Referral hospital in Lobatse).

**Sample size**

We have calculated sample size based on the finite formula below, using 5% as margin of error, 95% confidence interval, 50% population proportion, and population size of 2 million for Botswana regarding the general public, while for outpatients we used based on 1/4 of the general population.


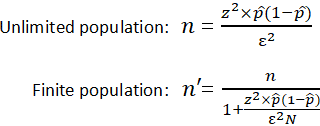


Where **z** is the z score, **ε** is the margin of error, **N** is the population size, and **p̂** is the population proportion

For the public, it gave us 385 as the sample size. Since we had 15 characteristics or subgroups classified into 3 categories (sociodemographic, self-reported stroke risk factors, and calculated stroke risk factors). Dividing 15 by 3 gave us 5. Therefore, we multiplied sample size 385 by 5 which gave 1925 as the sample size. For outpatient population size, we calculated 1/4 of the general population sample size which gave 481.

~~We did not calculate sample size since there are no studies with data on prevalence and/or incidence of MI in Botswana that could have helped us calculate it. Therefore, we aimed to recruit at least 70% of those targeted during data collection:~~ ~~at the same time taking into consideration our subgroups~~.

**Pilot study**

This was performed on the 10-13th April 2018 in Southeast District, Otse village, where 36 respondents comprising general public and outpatients were interviewed.

| **eFigure 1b. Knowledge of myocardial infarction symptoms and risk factors** |
| --- |

**Q1. Patient sociodemographic factors**

| **Gender** | \| **M** \| **F** \| \| --- \| --- \| | **Medical insurance** | \| **Yes** \| **No** \| \| --- \| --- \| |
| --- | --- | --- | --- | --- | --- | --- | --- |
| **Age (years)** |  | **Residing/working together** | \| **Yes** \| **No** \| \| --- \| --- \| |
| **Education** | \| **Primary** \| **Secondary** \| \| --- \| --- \| \| **Tertiary** \| **None** \| | **Marital status** | \| **Married** \| **Single** \| \| --- \| --- \| \| **Cohabiting** \| **Widowed/divorced** \| |
| **Location** | \| **Scot LH** \| **Moch V** \| \| --- \| --- \| \| **Phaph C** \| **Mathub** \| \| **Phuth C** \| **DRMH** \| \| **Sbrana** \| **Mathub C** \| \| **Nko C** \| **Moleps** \| | **If Gabz, select:**  **PE…**  **NBT…**  **BB…** |  |

**Scot LH=Scottish Livingstone Hospital Phaph C=Phaphane clinic**

**Mathub =Mathubudukwane village DRMH =Deborah Retief Memorial Hospital**

**Gabz= Gaborone city Moch=Mochudi village PE=Phakalne or Extension**

**Sbrana=Sbrana Psychiatric Referral Hospital Nko C=Nkoyaphiri clinic**

**Puth C=Phuthadikobo clinic Moleps=Molepolole village BB=Blocks or Broadhurst**

**NBT=Old Naledi, Bontleng or Tsolamosese Mathub C=Mathubudukwane clinic**

| **Q2:** Can you name symptoms of myocardial infarction (heart attack)*? (open-ended question)*  ***DO NOT MENTION THEM TO RESPONDENTS*** | |  |
| --- | --- | --- |
|  | Spontaneously recalled? |  |
| **Shortness of breath** | \| **yes** \| **no** \| \| --- \| --- \| |  |
| **Fanting/ dizziness** | \| yes \| no \| \| --- \| --- \| |  |
| **Central chest pain** | \| **yes** \| **no** \| \| --- \| --- \| |  |
| **Arm pain/ numbness** | \| yes \| no \| \| --- \| --- \| |  |
| **Sweating and clammy skin** | \| **yes** \| **no** \| \| --- \| --- \| |  |
| **Nausea** | \| yes \| no \| \| --- \| --- \| |  |
| **Feeling sick or looking pallor on the skin** | \| **yes** \| **no** \| \| --- \| --- \| |  |
| **Neck or jaw pain radiating from chest** | \| **Yes** \| **No** \| \| --- \| --- \| | |
| **Others:**  **………………….**  **…………………..** | \| **Yes** \| **No** \| \| --- \| --- \| \| **Yes** \| **No** \| | |

| **Q3**: Can you name conditions or lifestyle habits that may predispose to heart attack? (*open-ended question*)  ***DO NOT MENTION THEM TO THE RESPONDENTS*** | |
| --- | --- |
|  | Spontaneously recalled? |
| **Hypertension** | \| **yes** \|  \| **no** \|  \| \| --- \| --- \| --- \| --- \| |
| **Dyslipidemia** | \| yes \|  \| no \|  \| \| --- \| --- \| --- \| --- \| |
| **Diabetes** | \| **yes** \|  \| **no** \|  \| \| --- \| --- \| --- \| --- \| |
| **Obesity** | \| yes \|  \| no \|  \| \| --- \| --- \| --- \| --- \| |
| **Smoking** | \| **yes** \|  \| **no** \|  \| \| --- \| --- \| --- \| --- \| |
| **Sedentary lifestyle** | \| yes \|  \| no \|  \| \| --- \| --- \| --- \| --- \| |
| **Heavy alcohol intake** | \| **yes** \|  \| **no** \|  \| \| --- \| --- \| --- \| --- \| |
| **Previous stroke** | \| yes \|  \| no \|  \| \| --- \| --- \| --- \| --- \| |
| **Heart diseases** | \| **yes** \|  \| **no** \|  \| \| --- \| --- \| --- \| --- \| |
| **Family history of stroke/heart diseases** | \| yes \|  \| no \|  \| \| --- \| --- \| --- \| --- \| |
| **Others:**  ………………………….  …………………………. | \| **yes** \|  \| **no** \|  \| \| --- \| --- \| --- \| --- \| \| yes \|  \| no \|  \| |

**Q4a. Self-reporting (or medical reports). Do you have any stroke risk factors?**

|  | **Medical records** | **Self-reported** |  | **Medical records** | **Self-reported** |
| --- | --- | --- | --- | --- | --- |
| **Hypertension** | \| **Yes** \| **No** \| \| --- \| --- \| | \| **Yes** \| **No** \| \| --- \| --- \| | **Sedentary lifestyle** | \| **Yes** \| **No** \| \| --- \| --- \| | \| **Yes** \| **No** \| \| --- \| --- \| |
| **Diabetes** | \| **Yes** \| **No** \| \| --- \| --- \| | \| **Yes** \| **No** \| \| --- \| --- \| | **Smoking** | \| **Yes** \| **No** \| **Ex** \| \| --- \| --- \| --- \| | \| **Yes** \| **No** \| **Ex** \| \| --- \| --- \| --- \| |
| **Dyslipidemia** | \| **Yes** \| **No** \| \| --- \| --- \| | \| **Yes** \| **No** \| \| --- \| --- \| | **Heavy alcohol drinking** | \| **Yes** \| **No** \| **Ex** \| \| --- \| --- \| --- \| | \| **Yes** \| **No** \| **Ex** \| \| --- \| --- \| --- \| |
| **Heart diseases** | \| **Yes** \| **No** \| \| --- \| --- \| |  | **Previous stroke** | \| **Yes** \| **No** \| \| --- \| --- \| | \| **Yes** \| **No** \| \| --- \| --- \| |
| **Family history of stroke** | \| **Yes** \| **No** \| \| --- \| --- \| | \| **Yes** \| **No** \| \| --- \| --- \| | **Family history of both stroke and heart diseases** | \| **Yes** \| **No** \| \| --- \| --- \| | \| **Yes** \| **No** \| \| --- \| --- \| |
| **Family history of heart diseases** | \| **Yes** \| **No** \| \| --- \| --- \| | \| **Yes** \| **No** \| \| --- \| --- \| | **Obesity** | \| **Yes** \| **No** \| \| --- \| --- \| | \| **Yes** \| **No** \| \| --- \| --- \| |
| **HIV/AIDS**  **Other risk factors:**  **1……………**  **2……………**  **3……………** | \| **Yes** \| **No** \| \| --- \| --- \|  \| **Yes** \| **No** \| \| --- \| --- \| \| **Yes** \| **No** \| \| **Yes** \| **No** \| | \| **Yes** \| **No** \| \| --- \| --- \|  \| **Yes** \| **No** \| \| --- \| --- \| \| **Yes** \| **No** \| \| **Yes** \| **No** \| | **Psychiatric disease**  **In case yes, which ones do you have?**  **…………………**  **………………….** | \| **Yes** \| **No** \| \| --- \| --- \|  \| **Yes** \| **No** \| \| --- \| --- \| \| **Yes** \| **No** \| | \| **Yes** \| **No** \| \| --- \| --- \|  \| **Yes** \| **No** \| \| --- \| --- \| \| **Yes** \| **No** \| |

| **Do you do any physical activity?** | \| **Yes** \| **No** \| \| --- \| --- \| |
| --- | --- | --- | --- |
| **In case yes, what type of physical activities do you do?** | \| **1.** \| **2.** \| \| --- \| --- \| \| **3.** \| **4.** \| |
| **How will you grade the intensity of your physical activity?** | \| **1. Inactive** \| **2. Low** \| \| --- \| --- \| \| **3. Moderate** \| **4. High** \| \| **5. No idea** \|  \| |

| **What do you think of your weight?** | \| **No idea** \| **Normal** \| **Obese** \| \| --- \| --- \| --- \| | \| **Underweight** \| **Overweight** \| \| --- \| --- \| |
| --- | --- | --- | --- | --- | --- | --- | --- |
| **Do you think you eat healthy?** | \| **Yes** \| **No** \| **No idea** \| \| --- \| --- \| --- \| | **Height 1 (cm):**  **Height 2 (cm):**  **Weight (kg):** |
