# Supplementary material for: Age and healthy lifestyle behavior’s disparities and similarities on knowledge of myocardial infarction symptoms and risk factors among public and outpatients in a resource-limited setting, cross-sectional study in greater Gaborone, Botswana
Source: BMC Cardiovasc Disord. 2024 Mar 4;24:140. doi: 10.1186/s12872-024-03792-4 (PMC10910839; doi:10.1186/s12872-024-03792-4)
Supplement: Supplementary file 5 — Supplementary Material 5. [file 12872_2024_3792_MOESM5_ESM.docx]

|  |  |  |  |  |  |  |  |  |
| --- | --- | --- | --- | --- | --- | --- | --- | --- |
| **eTable 1. Differences in acknowledging of own myocardial infarction risk factors between public and outpatients stratified by age** | | | | | | | | |
|  |  |  |  |  |  |  |  |  |
|  | Age | Total | Aware | Public | Aware | Outpatients | Aware |  |
|  | (years) |  |  |  |  |  |  |  |
|  |  | n | n(%) | n | n(%) | n | n(%) | *p** |
| **Self-reported respondents’ risk factors** | | | |  |  |  |  |  |
| Hypertension | All | 258 | 155(60.1) | 161 | 88(54.7) | 97 | 67(69.1) | 0.313 |
|  | 18-34 | 31 | 16(51.6) | 19 | 8(42.1) | 12 | 8(66.7) | 0.458 |
|  | 35-49 | 105 | 67(63.8) | 60 | 36(60.0) | 45 | 31(68.9) | 0.660 |
|  | >50 | 122 | 72(59.1) | 82 | 44(53.7) | 40 | 28(70.0) | 0.389 |
|  |  |  |  |  |  |  |  |  |
| Family history of heart disease/ stroke | All | 1954 | 388(19.9) | 1488 | 263(17.7) | 466 | 125(26.8) | 0.009 |
|  | 18-34 | 1026 | 198(19.3) | 823 | 158(19.2) | 203 | 40(19.7) | 0.893 |
|  | 35-49 | 662 | 145(21.9) | 483 | 81(16.8) | 179 | 64(35.8) | <0.001 |
|  | >50 | 266 | 45(16.9) | 182 | 24(13.2) | 84 | 21(25.0) | 0.048 |
|  |  |  |  |  |  |  |  |  |
| Smoking | All | 355 | 142(40.0) | 271 | 86(31.7) | 84 | 56(66.7) | 0.004 |
|  | 18-34 | 145 | 43(29.7) | 127 | 31(24.4) | 18 | 12(66.7) | 0.015 |
|  | 35-49 | 148 | 72(48.6) | 103 | 44(42.7) | 45 | 28(62.2) | 0.210 |
|  | >50 | 62 | 27(43.5) | 41 | 11(26.8) | 21 | 16(76.2) | 0.025 |
|  |  |  |  |  |  |  |  |  |
| Sedentary lifestyle | All | 1904 | 762(40.0) | 1568 | 588(37.5) | 336 | 174(51.8) | 0.012 |
|  | 18-34 | 951 | 395(41.5) | 827 | 326(39.4) | 124 | 69(55.6) | 0.035 |
|  | 35-49 | 636 | 272(42.8) | 500 | 199(39.8) | 136 | 73(53.7) | 0.074 |
|  | >50 | 317 | 95(30.0) | 241 | 63(26.1) | 76 | 32(42.1) | 0.059 |
|  |  |  |  |  |  |  |  |  |
| **BMI status** |  |  |  |  |  |  |  |  |
| Overweight | All | 539 | 363(67.3) | 451 | 304(67.4) | 88 | 59(67.0) | 0.979 |
|  | 18-34 | 222 | 154(69.4) | 193 | 131(67.9) | 29 | 23(79.3) | 0.605 |
|  | 35-49 | 226 | 156(69.0) | 189 | 132(69.8) | 37 | 24(64.9) | 0.796 |
|  | >50 | 91 | 53(58.2) | 69 | 41(59.4) | 22 | 12(54.5) | 0.834 |
|  |  |  |  |  |  |  |  |  |
| Obesity | All | 32 | 26(81.3) | 19 | 15(78.9) | 13 | 11(84.6) | 0.902 |
|  | 18-34 | 8 | 6(75.0) | 4 | 3(75.0) | 4 | 3(75.0) | 0.999 |
|  | 35-49 | 17 | 15(88.2) | 11 | 9(81.8) | 6 | 6(100.0) | 0.784 |
|  | >50 | 7 | 5(71.4) | 4 | 3(75.0) | 3 | 2(66.7) | 0.921 |
|  |  |  |  |  |  |  |  |  |
| **Calculated respondents’ risk factors** | | | |  |  |  |  |  |
| **BMI status** |  |  |  |  |  |  |  |  |
| Overweight | All | 742 | 534(72.0) | 628 | 442(70.4) | 114 | 92(80.7) | 0.409 |
|  | 18-34 | 327 | 231(70.6) | 287 | 196(68.3) | 40 | 35(87.5) | 0.319 |
|  | 35-49 | 302 | 220(72.8) | 247 | 179(72.5) | 55 | 41(74.5) | 0.902 |
|  | >50 | 113 | 83(73.5) | 94 | 67(71.3) | 19 | 16(84.2) | 0.656 |
|  |  |  |  |  |  |  |  |  |
| Obesity | All | 496 | 313(63.1) | 405 | 252(62.2) | 91 | 61(67.0) | 0.715 |
|  | 18-34 | 152 | 103(67.8) | 129 | 85(65.9) | 23 | 18(78.3) | 0.617 |
|  | 35-49 | 217 | 146(67.3) | 173 | 116(67.1) | 44 | 30(68.2) | 0.950 |
|  | >50 | 127 | 64(50.4) | 103 | 51(49.5) | 24 | 13(54.2) | 0.815 |
|  |  |  |  |  |  |  |  |  |
| *: awareness differences between public and outpatients, calculated using chi-squared | | | | | |  |  |  |
|  |  |  |  |  |  |  |  |  |
