# Supplementary material for: Age and healthy lifestyle behavior’s disparities and similarities on knowledge of myocardial infarction symptoms and risk factors among public and outpatients in a resource-limited setting, cross-sectional study in greater Gaborone, Botswana
Source: BMC Cardiovasc Disord. 2024 Mar 4;24:140. doi: 10.1186/s12872-024-03792-4 (PMC10910839; doi:10.1186/s12872-024-03792-4)
Supplement: Supplementary file 6 — Supplementary Material 6. [file 12872_2024_3792_MOESM6_ESM.docx]

|  |  |  |  |  |  |
| --- | --- | --- | --- | --- | --- |
| **eTable 2. Awareness comparison of myocardial infarction risk factors among respondents with and without myocardial infarction risk factors stratified by age** | | | | | |
|  |  |  |  |  |  |
|  | Age | Aware | Unaware |  |  |
|  | (years) | n(%) | n(%) | *p* | OR |
| **TOTAL RESPONDENTS** | | |  |  |  |
| **Self-reported risk factors** | | | |  |  |
| **Hypertension** |  |  |  |  |  |
| Yes | All | 155 | 103 | 0.358 |  |
| No |  | 1547 | 909 |  |  |
| Yes | 18-34 | 16 | 15 | 0.273 |  |
| No |  | 868 | 548 |  |  |
| Yes | 35-49 | 67 | 38 | 0.543 |  |
| No |  | 527 | 262 |  |  |
| Yes | >50 | 72 | 50 | 0.777 |  |
| No |  | 152 | 99 |  |  |
| **Family history of stroke/heart diseases** | | | |  |  |
| Yes | All | 388 | 1566 | <0.001 | 2.4 |
| No |  | 70 | 690 |  |  |
| Yes | 18-34 | 198 | 828 | <0.001 | 2.8 |
| No |  | 37 | 384 |  |  |
| Yes | 35-49 | 145 | 517 | <0.001 | 2.2 |
| No |  | 27 | 205 |  |  |
| Yes | >50 | 45 | 221 | 0.004 | 3.4 |
| No |  | 6 | 101 |  |  |
| **Smoking** |  |  |  |  |  |
| Yes | All | 142 | 213 | 0.004 | 0.7 |
| No |  | 1137 | 1222 |  |  |
| Yes | 18-34 | 43 | 102 | <0.001 | 0.5 |
| No |  | 613 | 689 |  |  |
| Yes | 35-49 | 72 | 76 | 0.386 |  |
| No |  | 392 | 354 |  |  |
| Yes | >50 | 27 | 35 | 0.862 |  |
| No |  | 132 | 179 |  |  |
| **Sedentary lifestyle** |  |  |  |  |  |
| Yes | All | 762 | 1142 | 0.015 | 0.8 |
| No |  | 365 | 445 |  |  |
| Yes | 18-34 | 395 | 556 | 0.823 |  |
| No |  | 203 | 293 |  |  |
| Yes | 35-49 | 272 | 364 | 0.007 | 0.7 |
| No |  | 136 | 122 |  |  |
| Yes | >50 | 95 | 222 | 0.015 | 0.5 |
| No |  | 26 | 30 |  |  |
| **Overweight** |  |  |  |  |  |
| Yes | All | 363 | 176 | 0.372 |  |
| No |  | 1508 | 667 |  |  |
| Yes | 18-34 | 154 | 68 | 0.584 |  |
| No |  | 872 | 353 |  |  |
| Yes | 35-49 | 156 | 70 | 0.502 |  |
| No |  | 474 | 190 |  |  |
| Yes | >50 | 53 | 38 | 0.888 |  |
| No |  | 162 | 120 |  |  |
| **Obese** |  |  |  |  |  |
| Yes | All | 26 | 6 | *0.177 |  |
| No |  | 1845 | 837 |  |  |
| Yes | 18-34 | 6 | 2 | *0.999 |  |
| No |  | 1020 | 419 |  |  |
| Yes | 35-49 | 15 | 2 | *0.105 |  |
| No |  | 615 | 262 |  |  |
| Yes | >50 | 5 | 2 | *0.704 |  |
| No |  | 210 | 156 |  |  |
| **Calculated risk factors** | | | |  |  |
| **Overweight** |  |  |  |  |  |
| Yes | All | 534 | 208 | 0.036 | 1.2 |
| No |  | 1337 | 635 |  |  |
| Yes | 18-34 | 231 | 96 | 0.920 |  |
| No |  | 795 | 325 |  |  |
| Yes | 35-49 | 220 | 82 | 0.265 |  |
| No |  | 410 | 182 |  |  |
| Yes | >50 | 83 | 30 | <0.001 | 2.7 |
| No |  | 132 | 128 |  |  |
| **Obese** |  |  |  |  |  |
| Yes | All | 313 | 183 | 0.002 | 0.7 |
| No |  | 1558 | 660 |  |  |
| Yes | 18-34 | 103 | 49 | 0.368 |  |
| No |  | 923 | 372 |  |  |
| Yes | 35-49 | 146 | 71 | 0.237 |  |
| No |  | 484 | 193 |  |  |
| Yes | >50 | 64 | 63 | 0.042 | 0.6 |
| No |  | 151 | 95 |  |  |
|  |  |  |  |  |  |
| **PUBLIC** |  |  |  |  |  |
| **Self-reported risk factors** | | | |  |  |
| **Hypertension** |  |  |  |  |  |
| Yes | All | 88 | 73 | 0.278 |  |
| No |  | 1203 | 835 |  |  |
| Yes | 18-34 | 8 | 11 | 0.155 |  |
| No |  | 711 | 508 |  |  |
| Yes | 35-49 | 36 | 24 | 0.752 |  |
| No |  | 387 | 236 |  |  |
| Yes | >50 | 44 | 38 | 0.999 |  |
| No |  | 105 | 91 |  |  |
| **Family history of stroke/heart diseases** | | | |  |  |
| Yes | All | 263 | 1225 | <0.001 | 2.4 |
| No |  | 58 | 653 |  |  |
| Yes | 18-34 | 158 | 665 | <0.001 | 2.6 |
| No |  | 35 | 380 |  |  |
| Yes | 35-49 | 81 | 402 | 0.014 | 1.9 |
| No |  | 19 | 181 |  |  |
| Yes | >50 | 24 | 158 | *0.020 | 3.5 |
| No |  | 4 | 92 |  |  |
| **Smoking** |  |  |  |  |  |
| Yes | All | 86 | 185 | <0.001 | 0.6 |
| No |  | 841 | 1087 |  |  |
| Yes | 18-34 | 31 | 96 | <0.001 | 0.4 |
| No |  | 494 | 617 |  |  |
| Yes | 35-49 | 44 | 59 | 0.512 |  |
| No |  | 268 | 312 |  |  |
| Yes | >50 | 11 | 30 | 0.410 |  |
| No |  | 79 | 158 |  |  |
| **Sedentary lifestyle** |  |  |  |  |  |
| Yes | All | 588 | 980 | <0.001 | 0.7 |
| No |  | 296 | 335 |  |  |
| Yes | 18-34 | 326 | 501 | 0.072 |  |
| No |  | 184 | 227 |  |  |
| Yes | 35-49 | 199 | 301 | 0.002 | 0.6 |
| No |  | 97 | 86 |  |  |
| Yes | >50 | 63 | 178 | 0.070 |  |
| No |  | 15 | 22 |  |  |
| **Overweight** |  |  |  |  |  |
| Yes | All | 304 | 147 | 0.796 |  |
| No |  | 1167 | 581 |  |  |
| Yes | 18-34 | 131 | 62 | 0.841 |  |
| No |  | 717 | 328 |  |  |
| Yes | 35-49 | 132 | 57 | 0.920 |  |
| No |  | 343 | 151 |  |  |
| Yes | >50 | 41 | 28 | 0.235 |  |
| No |  | 107 | 102 |  |  |
| **Obese** |  |  |  |  |  |
| Yes | All | 15 | 4 | *0.333 |  |
| No |  | 1456 | 724 |  |  |
| Yes | 18-34 | 3 | 1 | *0.999 |  |
| No |  | 845 | 389 |  |  |
| Yes | 35-49 | 9 | 2 | *0.518 |  |
| No |  | 466 | 206 |  |  |
| Yes | >50 | 3 | 1 | *0.625 |  |
| No |  | 145 | 129 |  |  |
| **Calculated risk factors** | | | |  |  |
| **Overweight** |  |  |  |  |  |
| Yes | All | 442 | 186 | 0.028 | 1.3 |
| No |  | 1029 | 542 |  |  |
| Yes | 18-34 | 196 | 91 | 0.920 |  |
| No |  | 652 | 299 |  |  |
| Yes | 35-49 | 179 | 68 | 0.212 |  |
| No |  | 296 | 140 |  |  |
| Yes | >50 | 67 | 27 | <0.001 | 3.2 |
| No |  | 81 | 103 |  |  |
| **Obese** |  |  |  |  |  |
| Yes | All | 252 | 153 | 0.027 | 0.8 |
| No |  | 1219 | 575 |  |  |
| Yes | 18-34 | 85 | 44 | 0.502 |  |
| No |  | 763 | 346 |  |  |
| Yes | 35-49 | 116 | 57 | 0.410 |  |
| No |  | 359 | 151 |  |  |
| Yes | >50 | 51 | 52 | 0.340 |  |
| No |  | 97 | 78 |  |  |
|  |  |  |  |  |  |
| **OUTPATIENTS** |  |  |  |  |  |
| **Self-reported risk factors** | | | |  |  |
| **Hypertension** |  |  |  |  |  |
| Yes | All | 67 | 30 | 0.003 | 0.5 |
| No |  | 344 | 74 |  |  |
| Yes | 18-34 | 8 | 4 | *0.467 |  |
| No |  | 157 | 40 |  |  |
| Yes | 35-49 | 31 | 14 | 0.019 | 0.4 |
| No |  | 140 | 26 |  |  |
| Yes | >50 | 28 | 12 | 0.068 |  |
| No |  | 47 | 8 |  |  |
| **Family history of stroke/heart diseases** | | | |  |  |
| Yes | All | 125 | 341 | 0.725 |  |
| No |  | 12 | 37 |  |  |
| Yes | 18-34 | 40 | 163 | *0.603 |  |
| No |  | 2 | 4 |  |  |
| Yes | 35-49 | 64 | 115 | 0.237 |  |
| No |  | 8 | 24 |  |  |
| Yes | >50 | 21 | 63 | *0.729 |  |
| No |  | 2 | 9 |  |  |
| **Smoking** |  |  |  |  |  |
| Yes | All | 56 | 28 | 0.717 |  |
| No |  | 296 | 135 |  |  |
| Yes | 18-34 | 12 | 6 | 0.718 |  |
| No |  | 119 | 72 |  |  |
| Yes | 35-49 | 28 | 17 | 0.098 |  |
| No |  | 124 | 42 |  |  |
| Yes | >50 | 16 | 5 | *0.786 |  |
| No |  | 53 | 21 |  |  |
| **Sedentary lifestyle** |  |  |  |  |  |
| Yes | All | 174 | 162 | 0.004 | 1.7 |
| No |  | 69 | 110 |  |  |
| Yes | 18-34 | 69 | 55 | <0.001 | 4.4 |
| No |  | 19 | 66 |  |  |
| Yes | 35-49 | 73 | 63 | 0.823 |  |
| No |  | 39 | 36 |  |  |
| Yes | >50 | 32 | 44 | 0.216 |  |
| No |  | 11 | 8 |  |  |
| **Overweight** |  |  |  |  |  |
| Yes | All | 59 | 29 | 0.009 | 0.5 |
| No |  | 341 | 86 |  |  |
| Yes | 18-34 | 23 | 6 | 0.339 |  |
| No |  | 155 | 25 |  |  |
| Yes | 35-49 | 24 | 13 | 0.192 |  |
| No |  | 131 | 43 |  |  |
| Yes | >50 | 12 | 10 | 0.060 |  |
| No |  | 55 | 18 |  |  |
| **Obese** |  |  |  |  |  |
| Yes | All | 11 | 2 | *0.742 |  |
| No |  | 389 | 113 |  |  |
| Yes | 18-34 | 3 | 1 | *0.999 |  |
| No |  | 175 | 30 |  |  |
| Yes | 35-49 | 6 | 0 | 0.135 |  |
| No |  | 149 | 56 |  |  |
| Yes | >50 | 2 | 1 | *0.999 |  |
| No |  | 65 | 27 |  |  |
| **Calculated risk factors** | | | |  |  |
| **Overweight** |  |  |  |  |  |
| Yes | All | 92 | 22 | 0.378 |  |
| No |  | 308 | 93 |  |  |
| Yes | 18-34 | 35 | 5 | *0.806 |  |
| No |  | 143 | 26 |  |  |
| Yes | 35-49 | 41 | 14 | 0.840 |  |
| No |  | 114 | 42 |  |  |
| Yes | >50 | 16 | 3 | *0.171 |  |
| No |  | 51 | 25 |  |  |
| **Obese** |  |  |  |  |  |
| Yes | All | 61 | 30 | 0.007 | 0.5 |
| No |  | 339 | 85 |  |  |
| Yes | 18-34 | 18 | 5 | *0.350 |  |
| No |  | 160 | 26 |  |  |
| Yes | 35-49 | 30 | 14 | 0.374 |  |
| No |  | 125 | 42 |  |  |
| Yes | >50 | 13 | 11 | 0.042 | 0.4 |
| No |  | 54 | 17 |  |  |
|  |  |  |  |  |  |
| *: used Fisher's exact, OR: odds ratio | | | | |  |
|  |  |  |  |  |  |
