# Supplementary material for: Age and healthy lifestyle behavior’s disparities and similarities on knowledge of myocardial infarction symptoms and risk factors among public and outpatients in a resource-limited setting, cross-sectional study in greater Gaborone, Botswana
Source: BMC Cardiovasc Disord. 2024 Mar 4;24:140. doi: 10.1186/s12872-024-03792-4 (PMC10910839; doi:10.1186/s12872-024-03792-4)
Supplement: Supplementary file 7 — Supplementary Material 7. [file 12872_2024_3792_MOESM7_ESM.docx]

|  |  |  |  |  |
| --- | --- | --- | --- | --- |
| **eTable 3. ANOVA-Sociodemographic and MI risk factors association with MI mean knowledge scores among respondents** | | | | |
|  |  |  |  |  |
|  |  |  |  |  |
|  | **Myocardial infarction symptoms** | | **Myocardial infarction risk factors** | |
|  | Public | Outpatient | Public | Outpatient |
|  | Mean ±SD | Mean ±SD | Mean ±SD | Mean ±SD |
| **Sociodemographic factors** | |  |  |  |
| **Respondents' type** | |  |  |  |
| Public | 2.80±2.54 | NA | 3.82±3.07 | NA |
| Outpatients | NA | 3.49±2.84 | NA | 5.33±3.22 |
| ***p*** | NA | NA | NA | NA |
|  |  |  |  |  |
| **Gender** |  |  |  |  |
| Female | 2.77±2.54 | 3.38±2.90 | 3.72±3.02 | 5.32±3.23 |
| Male | 2.85±2.54 | 3.61±2.77 | 3.96±3.12 | 5.33±3.22 |
| ***p*** | 0.427 | 0.156* | 0.074 | 0.835 |
|  |  |  |  |  |
| **Age (years)** |  |  |  |  |
| 1. 18-34 | 2.94±2.56 | 3.27±2.64 | 3.91±3.06 | 4.89±2.90 |
| 2. 35-49 | 2.85±2.49 | 3.79±3.07 | 4.01±3.09 | 5.76±3.43 |
| 3. >50 | 2.08±2.43 | 3.28±2.67 | 2.95±2.89 | 5.34±3.32 |
| ***p*** | <0.001*; 1 vs 3<0.001, 2 vs 3<0.001 | 0.684* | <0.001; 1 vs 3<0.001, 2 vs 3<0.001 | 0.755* |
| **η_p_^2^** | 0.012 |  | 0.012 |  |
|  |  |  |  |  |
| **Education** |  |  |  |  |
| 1. Primary/ unspecified | 1.86±2.45 | 2.85±2.81 | 2.51±2.99 | 4.40±3.68 |
| 2. Secondary | 2.76±2.62 | 3.09±2.79 | 3.70±3.09 | 4.91±3.28 |
| 3. Tertiary | 3.12±2.39 | 4.15±2.79 | 4.35±2.93 | 6.09±2.86 |
| ***p*** | <0.001*; 1 vs 2<0.001, 1 vs 3<0.001, 2 vs 3=0.005 | <0.001*; 1 vs 3=0.005, 2 vs 3<0.001 | <0.001; 1 vs 2<0.001, 1 vs 3<0.001, 2 vs 3<0.001 | <0.001*; 1 vs 3=0.006, 2 vs 3<0.001 |
| **η_p_^2^** | 0.023 | 0.041 | 0.034 | 0.041 |
|  |  |  |  |  |
| **Medical insurance** | |  |  |  |
| No | 2.78±2.56 | 3.26±2.80 | 3.77±3.10 | 5.05±3.20 |
| Yes | 2.99±2.38 | 4.43±2.81 | 4.17±2.77 | 6.55±3.03 |
| ***p*** | 0.166* | <0.001 | 0.028* | <0.001* |
| ***d*** |  | 0.383 | 0.130 | 0.426 |
|  |  |  |  |  |
| **Residing/working together** | |  |  |  |
| No | 2.34±2.19 | 3.02±2.71 | 3.49±2.81 | 5.14±3.17 |
| Yes | 3.86±2.93 | 4.32±2.88 | 4.58±3.46 | 5.66±3.30 |
| ***p*** | <0.001* | <0.001* | <0.001* | <0.001* |
| ***d*** | 0.600 | 0.528 | 0.358 | 0.207 |
|  |  |  |  |  |
| **Marital status** |  |  |  |  |
| No | 2.78±2.59 | 3.27±2.78 | 3.85±3.09 | 5.01±3.17 |
| Yes | 2.86±2.40 | 3.96±2.90 | 3.76±3.02 | 6.04±3.24 |
| ***p*** | 0.483* | 0.080 | 0.543 | 0.012 |
| ***d*** |  |  |  | 0.241 |
|  |  |  |  |  |
| **Respodents' self-reported risk factors** | |  |  |  |
| **History of hypertension** | |  |  |  |
| No/ unspecified | 2.86±2.54 | 3.78±2.79 | 3.87±3.06 | 5.61±3.18 |
| Yes | 2.08±2.42 | 2.23±2.70 | 3.19±3.05 | 4.12±3.14 |
| ***p*** | <0.001 | <0.001* | 0.006 | <0.001* |
| ***d*** | 0.308 | 0.659 | 0.224 | 0.510 |
|  |  |  |  |  |
| **History of CVDS** | |  |  |  |
| No | 2.79±2.53 | 3.58±2.83 | 3.81±3.07 | 5.41±3.23 |
| Yes | 3.27±2.76 | 2.79±2.81 | 4.23±3.08 | 4.67±3.12 |
| ***p*** | 0.117 | 0.020 | 0.259 | 0.092 |
| ***d*** |  | 0.316 |  |  |
|  |  |  |  |  |
| **Family history of stroke/heart diseases** | | |  |  |
| 1. None/ unspecified | 2.04±2.28 | 2.06±3.09 | 2.52±2.76 | 3.33±3.99 |
| 2. Both stroke and heart diseases | 3.35±2.61 | 4.69±2.95 | 4.75±3.10 | 6.51±3.19 |
| 3. Heart diseases | 2.74±2.41 | 2.43±2.27 | 3.92±2.80 | 4.29±2.58 |
| 4. Stroke | 3.83±2.68 | 4.30±2.40 | 5.07±3.11 | 6.44±2.81 |
| ***p*** | <0.001*; 1 vs 2<0.001, 1 vs 3<0.001, 1 vs 4<0.001, 2 vs 3<0.001, 3 vs 4<0.001 | <0.001*; 1 vs 2<0.001, 1 vs 4<0.001, 2 vs 3<0.001, 3 vs 4<0.001 | <0.001*; 1 vs 2<0.001, 1 vs 3<0.001, 1 vs 4<0.001, 2 vs 3<0.001, 3 vs 4<0.001 | <0.001*; 1 vs 2<0.001, 1 vs 4<0.001, 2 vs3 <0.001, 3 vs 4<0.001 |
| **η_p_^2^** | 0.062 | 0.153 | 0.103 | 0.155 |
|  |  |  |  |  |
| **Smoking** |  |  |  |  |
| 1. None/ unspecified | 2.83±2.53 | 3.39±2.75 | 3.88±3.04 | 5.23±3.03 |
| 2. Current | 2.46±2.51 | 3.55±3.13 | 3.27±3.07 | 5.25±3.83 |
| 3. Former | 3.78±2.84 | 4.95±3.09 | 4.95±3.53 | 7.32±3.64 |
| ***p*** | 0.004; 2 vs 3=0.007 | 0.152* | <0.001; 1 vs 2=0.007, 2 vs 3<0.004 | 0.165* |
| **η_p_^2^** | 0.005 |  | 0.007 |  |
|  |  |  |  |  |
| **Alcohol consumption status** | |  |  |  |
| 1. None/ unspecified | 2.93±2.60 | 3.32±2.74 | 3.99±3.14 | 5.06±3.14 |
| 2. Current | 2.42±2.32 | 3.88±2.93 | 3.32±2.80 | 5.86±3.32 |
| 3. Former | 3.27±2.61 | 3.58±3.44 | 4.39±3.19 | 6.00±3.50 |
| ***p*** | 0.001*; 1 vs 2<0.001 | 0.389* | 0.001*; 1 vs 2<0.001 | 0.365* |
| **η_p_^2^** | 0.009 |  | 0.010 |  |
|  |  |  |  |  |
| **Healthy dietary status** | |  |  |  |
| No/ unspecified | 2.73±2.65 | 3.38±3.31 | 3.58±3.23 | 5.15±3.65 |
| Yes | 2.85±2.46 | 3.52±2.66 | 3.99±2.93 | 5.38±3.07 |
| ***p*** | 0.275* | 0.025* | <0.002* | 0.051* |
| ***d*** |  | 0.263 | 0.135 |  |
|  |  |  |  |  |
| **History of HIV/AIDS** | |  |  |  |
| No/unspecified | 2.77±2.58 | 2.71±2.76 | 3.68±3.07 | 4.32±3.13 |
| Yes | 3.04±2.22 | 4.57±2.58 | 4.82±2.83 | 6.74±2.80 |
| ***p*** | 0.069* | <0.001* | <0.001 | <0.001* |
| ***d*** |  | 0.650 | 0.372 | 0.706 |
|  |  |  |  |  |
| **History of psychiatric diseases** | |  |  |  |
| No | NA | 3.87±2.96 | NA | 5.92±3.23 |
| Yes | NA | 1.67±0.89 | NA | 2.53±0.82 |
| ***p*** | NA | <0.001* | NA | <0.001* |
| ***d*** | NA | 0.464 | NA | 0.778 |
|  |  |  |  |  |
| **Physical intensity levels** | |  |  |  |
| 1. None/ unspecified | 2.67±2.54 | 3.66±2.92 | 3.63±3.05 | 5.59±3.21 |
| 2. Light | 2.76±2.57 | 2.96±2.98 | 4.06±3.03 | 5.10±3.37 |
| 3. Moderate | 3.34±2.45 | 3.08±2.37 | 4.53±2.99 | 4.64±3.06 |
| 4. High | 2.90±2.57 | 4.61±3.24 | 3.65±3.27 | 5.72±3.69 |
| ***p*** | 0.001; 1 vs 3<0.001 | 0.161* | <0.001; 1 vs 3<0.001 | 0.178* |
| **η_p_^2^** | 0.010 |  | 0.013 |  |
|  |  |  |  |  |
| **Respondents’ calculated risk factors** | | |  |  |
| **BMI status** |  |  |  |  |
| 1. Underweight | 2.72±2.60 | 4.08±3.34 | 3.78±3.38 | 5.50±3.87 |
| 2. Normal, unknown | 2.95±2.66 | 3.79±2.78 | 3.96±3.11 | 5.52±3.19 |
| 3. Overweight | 2.87±2.43 | 3.38±2.82 | 3.94±3.03 | 5.40±3.14 |
| 4. Obesity | 2.34±2.30 | 2.56±2.83 | 3.27±2.86 | 4.56±3.29 |
| ***p*** | <0.001*; 2 vs 4<0.001, 3 vs 4=0.002 | 0.001*; 2 vs 4<0.001 | <0.001*; 2 vs 4<0.001, 3 vs 4=0.002 | <0.001*; 2 vs 4<0.001 |
| **η_p_^2^** | 0.008 | 0.038 | 0.007 | 0.038 |

CVDS: cardiovascular diseases (dyslipidemia, diabetes, stroke, or heart diseases),

BMI: body mass index, NA: not applicable, MI: myocardial infarction, *d*: Cohen’s d, ηp2: partial eta squared, *: equal variance not assumed
