# Supplementary material for: Age and healthy lifestyle behavior’s disparities and similarities on knowledge of myocardial infarction symptoms and risk factors among public and outpatients in a resource-limited setting, cross-sectional study in greater Gaborone, Botswana
Source: BMC Cardiovasc Disord. 2024 Mar 4;24:140. doi: 10.1186/s12872-024-03792-4 (PMC10910839; doi:10.1186/s12872-024-03792-4)
Supplement: Supplementary file 8 — Supplementary Material 8. [file 12872_2024_3792_MOESM8_ESM.docx]

|  | |  | |  | | |  |  |
| --- | --- | --- | --- | --- | --- | --- | --- | --- |
| **eTable 4. Factorial ANOVA-Association of healthy lifestyle behaviours adjusted sociodemographic and MI risk factors with MI knowledge**  **scores among respondents** | | | | | | | | |
|  |  | |  | |  |  | | |
|  | **MI symptoms** | |  | | **MI risk factors** |  | | |
|  | Public | | Outpatient | | Public | Outpatient | | |
|  | Mean±SD | | Mean±SD | | Mean±SD | Mean±SD | | |
| **SOCIODEMOGRAPHIC FACTORS** |  | |  | |  |  | | |
| **Respondents' type** |  | |  | |  |  | | |
| Publicxlifestyle0 | 2.71±2.67 | | NA | | 3.53±3.29 | NA | | |
| Publicxlifestyle1 | 2.70±2.49 | | NA | | 3.76±2.93 | NA | | |
| Publicxlifestyle2 | 3.09±2.48 | | NA | | 4.23±3.04 | NA | | |
| Outpatientsx lifestyle0 | NA | | 3.64±3.39 | | NA | 5.49±3.52 | | |
| Outpatientsxlifestyle1 | NA | | 3.57±2.77 | | NA | 5.51±3.11 | | |
| Outpatientsxlifestyle3 | NA | | 3.32±2.69 | | NA | 5.03±3.25 | | |
|  |  | |  | |  |  | | |
| *Overall p* | NA | | NA | | NA | NA | | |
|  |  | |  | |  |  | | |
| **Age** |  | |  | |  |  | | |
| Lifestyle0x1 | 3.01±2.77 | | 4.03±3.38 | | 3.93±3.38 | 6.16±3.30 | | |
| Lifestyle0x2 | 2.50±2.41 | | 3.88±3.50 | | 3.22±3.03 | 5.88±3.27 | | |
| Lifestyle0x3 | 1.95±2.58 | | 2.53±3.18 | | 2.60±3.17 | 3.65±3.82 | | |
| Lifestyle1x1 | 2.85±2.51 | | 3.49±2.71 | | 3.82±2.94 | 5.31±2.84 | | |
| Lifestyle1x2 | 2.78±2.49 | | 3.75±3.05 | | 4.04±2.98 | 5.70±3.36 | | |
| Lifestyle1x3 | 1.99±2.28 | | 3.36±2.36 | | 2.93±2.62 | 5.53±3.12 | | |
| Lifestyle2x1 | 3.03±2.44 | | 2.70±2.11 | | 4.05±2.93 | 3.89±2.50 | | |
| Lifestyle2x2 | 3.31±2.51 | | 3.81±3.01 | | 4.73±3.17 | 5.78±3.59 | | |
| Lifestyle2x3 | 2.57±2.59 | | 3.64±2.91 | | 3.57±3.15 | 6.08±3.11 | | |
|  |  | |  | |  |  | | |
| *p* | LS0x1 vs LS0x3=0.002, d=0.006; LS1x1 vs LS1x3<0.001, d=0.006; LS1x2 vs LS1x3=0.005, d=0.006 | |  | | LS0x1 vs LS0x2=0.038, d=0.007, LS0x1 vs LS0x3=0.001, d=0.007; LS1x1 vs LS1x3=0.005, d=0.006; LS1x2 vs LS1x3<0.001, d=0.006; LS2x1 vs LS2x2=0.042, d=0.004 | LS0x1 vs LS0x3=0.005,  d=0.022;  LS0x2 vs LS0x3=0.016,  d=0.022 | | |
| *Overall p* | 0.188 | | 0.524 | | 0.010 | 0.002 | | |
| *Overall d* |  | |  | | 0.006 | 0.033 | | |
|  |  | |  | |  |  | | |
| **Gender** |  | |  | |  |  | | |
| Femalexlifestyle0 | 2.65±2.75 | | 3.25±3.35 | | 3.44±3.30 | 5.23±3.48 | | |
| Femalexlifestyle1 | 2.64±2.43 | | 3.42±2.88 | | 3.67±2.84 | 5.39±3.14 | | |
| Femalexlifestyle2 | 3.20±2.43 | | 3.40±2.61 | | 4.22±2.96 | 5.27±3.28 | | |
| Malexlifestyle0 | 2.82±2.51 | | 4.62±3.38 | | 3.71±3.26 | 6.14±3.60 | | |
| Malexlifestyle1 | 2.78±2.57 | | 3.80±2.58 | | 3.88±3.04 | 5.71±3.06 | | |
| Malexlifestyle2 | 2.98±2.52 | | 3.28±2.76 | | 4.24±3.13 | 4.88±3.23 | | |
| *p* |  | |  | |  |  | | |
| *Overall p* | 0.341 | | 0.125 | | 0.756 | 0.367 | | |
|  |  | |  | |  |  | | |
| **Education** |  | |  | |  |  | | |
| PrimaryxLifestyle0 | 2.15±2.65 | | 2.46±3.33 | | 2.70±3.13 | 3.62±3.93 | | |
| PrimaryxLifestyle1 | 1.79±2.40 | | 2.84±2.50 | | 2.50±2.96 | 4.84±3.58 | | |
| PrimaryxLifestyle2 | 1.22±1.79 | | 3.21±2.99 | | 1.97±2.72 | 4.36±3.78 | | |
| SecondaryxLifestyle0 | 2.51±2.64 | | 3.48±3.55 | | 3.27±3.21 | 5.36±3.76 | | |
| SecondaryxLifestyle1 | 2.70±2.52 | | 3.09±2.68 | | 3.74±2.92 | 4.96±3.09 | | |
| SecondaryxLifestyle2 | 3.24±2.77 | | 2.97±2.67 | | 4.15±3.22 | 4.71±3.36 | | |
| TertiaryxLifestyle0 | 3.36±2.61 | | 4.36±3.17 | | 4.45±3.31 | 6.50±2.66 | | |
| TertiaryxLifestyle1 | 2.99±2.41 | | 4.36±2.80 | | 4.16±2.82 | 6.38±2.81 | | |
| TertiaryxLifestyle2 | 3.16±2.21 | | 3.81±2.63 | | 4.52±2.83 | 5.58±2.94 | | |
|  |  | |  | |  |  | | |
| *p* | LS0xPrimary vs LS0xTertiary<0.001, d=0.009; LS0xSecondary vs LS0xTertiary<0.001, d=0.009; LS1xPrimary vs LS1xSecondary<0.001, d=0.010; LS1xPrimary vs LS1xTertiary<0.001, d=0.010; LS2xPrimary vs LS2xSecondary<0.001, d=0.009; LS2xPrimary vs LS2xTertiary<0.001, d=0.009 | | LS1xPrimary vs LS1xTertiary=0.027, d=0.027; LS1xSecondary vs LS1xTertiary=0.002, d=0.027 | | LS0xPrimay vs LS0xTertiary<0.001, d=0.012; LS0xSecondary vs LS0xTertiary<0.001, d=0.012; LS1xPrimary vs LS1xSecondary<0.001, d=0.013; LS1xPrimary vs LS1xTertiary<0.001, d=0.013; LS2xPrimary vs LS2xSecondary<0.001, d=0.010; LS2xPrimary vs LS2xTertiary<0.001, d=0.010 | LS0xPrimary vs LS0xTertiary  <0.001,  d=0.026;  LS1xSecondary vs  LS1xTertiary  =0.002, d=0.030;  LS1xPrimary vs LS1xTertiary  =0.008,  d=0.030 | | |
| *Overall p* | 0.008 | | 0.757 | | 0.046 | 0.540 | | |
| *Overall d* | 0.006 | |  | | 0.004 |  | | |
|  |  | |  | |  |  | | |
| **Medical Insurance** |  | |  | |  |  | | |
| NoxLifestyle0 | 2.66±2.66 | | 3.48±3.36 | | 3.47±3.23 | 5.32±3.48 | | |
| NoxLifestyle1 | 2.69±2.48 | | 3.37±2.76 | | 3.71±2.94 | 5.29±3.10 | | |
| NoxLifestyle2 | 3.11±2.57 | | 3.01±2.56 | | 4.26±3.15 | 4.57±3.17 | | |
| YesxLifestyle0 | 3.50±2.72 | | 5.80±3.49 | | 4.47±3.32 | 7.80±3.49 | | |
| YesxLifestyle1 | 2.83±2.53 | | 4.35±2.71 | | 4.14±2.77 | 6.41±3.00 | | |
| YesxLifestyle2 | 2.99±2.10 | | 4.38±2.87 | | 4.11±2.59 | 6.56±3.06 | | |
|  |  | |  | |  |  | | |
| *p* | LS0xNo vs LS0xYes =0.047, d=0.002 | | LS1xNo vs LS1xYes=0.022, d=0.010; LS2xNo vs LS2xYes=0.046, d=0.008 | |  | LS1xNo vs LS1xYes=0.037,  d=0.009; LS2xNo vs LS2xYes  =0.002, d=0.018 | | |
| *Overall p* | 0.155 | | 0.713 | | 0.127 | 0.603 | | |
|  |  | |  | |  |  | | |
| **Residing together** |  | |  | |  |  | | |
| NoxLifestyle0 | 2.11±2.13 | | 1.74±2.70 | | 2.85±2.71 | 3.85±3.30 | | |
| NoxLifestyle1 | 2.18±2.11 | | 3.01±2.53 | | 3.41±2.67 | 5.07±2.98 | | |
| NoxLifestyle2 | 2.85±2.31 | | 3.63±2.84 | | 4.24±2.98 | 5.86±3.26 | | |
| YesxLifestyle0 | 3.95±3.19 | | 6.93±1.30 | | 4.95±3.86 | 8.33±1.47 | | |
| YesxLifestyle1 | 3.91±2.85 | | 5.11±2.85 | | 4.56±3.31 | 6.73±3.16 | | |
| YesxLifestyle2 | 3.66±2.77 | | 2.98±2.49 | | 4.21±3.20 | 4.09±2.98 | | |
|  |  | |  | |  |  | | |
| *p* | LS0xNo vs LS0xYes<0.001, d=0.033; LS1xNo vs LS1xYes<0.001, d=0.049; LS2xNo vs LS2xYes<0.001, d=0.006 | | LS0xNo vs LS0xYes<0.001, d=0.119; LS1xNo vs LS1xYes<0.001, d=0.049 | | LS0xNo vs LS0xYes<0.001, d=0.028; LS1xNo vs LS1xYes<0.001, d=0.014 | LS0xNo vs LS0xYes<0.001,  d=0.066;  LS1xNo vs LS1xYes<0.001,  d=0.023; LS2xNo vs LS2xYes  =0.003, d=0.017 | | |
| *Overall p* | <0.001 | | <0.001 | | <0.001 | <0.001 | | |
| *Overall d* | 0.006 | | 0.096 | | 0.014 | 0.088 | | |
|  |  | |  | |  |  | | |
| **Marital status** |  | |  | |  |  | | |
| NoxLifestyle0 | 2.73±2.73 | | 3.76±3.41 | | 3.61±3.30 | 5.68±3.48 | | |
| NoxLifestyle1 | 2.72±2.58 | | 3.34±2.77 | | 3.83±3.01 | 5.21±3.07 | | |
| NoxLifestyle2 | 2.93±2.46 | | 3.03±2.55 | | 4.13±2.97 | 4.54±3.12 | | |
| YesxLifestyle0 | 2.65±2.49 | | 3.38±3.40 | | 3.33±3.26 | 5.08±3.64 | | |
| YesxLifestyle1 | 2.65±2.26 | | 4.01±2.74 | | 3.60±2.71 | 6.10±3.11 | | |
| YesxLifestyle2 | 3.46±2.49 | | 4.16±2.93 | | 4.48±3.22 | 6.41±3.23 | | |
|  |  | |  | |  |  | | |
| *p* | LS2xNo vs LS2xYes=0.025, d=0.002 | |  | |  | LS2xNo vs LS2xYes=0.003,  d=0.017 | | |
| *Overall p* | 0.087 | | 0.359 | | 0.186 | 0.060 | | |
| *Overall d* |  | |  | |  |  | | |
|  |  | |  | |  |  | | |
| **SELF-REPORTED RISK FACTORS** |  | |  | |  |  | | |
| **Hypertension** |  | |  | |  |  | | |
| NoxLifestyle0 | 2.79±2.69 | | 4.48±3.31 | | 3.60±3.31 | 6.44±3.29 | | |
| NoxLifestyle1 | 2.77±2.48 | | 3.89±2.74 | | 3.83±2.92 | 5.82±3.01 | | |
| NoxLifestyle2 | 3.10±2.47 | | 3.44±2.65 | | 4.21±3.03 | 5.10±3.28 | | |
| YesxLifestyle0 | 1.77±2.20 | | 1.64±2.72 | | 2.77±2.93 | 3.23±3.02 | | |
| YesxLifestyle1 | 1.82±2.37 | | 2.33±2.58 | | 2.78±2.89 | 4.31±3.22 | | |
| YesxLifestyle2 | 2.93±2.63 | | 2.54±2.95 | | 4.43±3.20 | 4.54±3.04 | | |
|  |  | |  | |  |  | | |
| *p* | LS0xNo vs LS0xYes=0.008, d=0.003; LS1xNo vs LS1xYes=0.002, d=0.004 | | LS0xNo vs LS0xYes<0.001, d=0.036; LS1xNo vs LS1xYes<0.001, d=0.030; LS2xNo vs LS2xYes=0.024, d=0.010 | | LS1xNo vs LS1xYes=0.004, d=0.004 | LS0xNo vs LS0xYes<0.001,  d=0.033; LS1xNo vs LS1x  Yes<0.001, d=0.024 | | |
| *Overall p* | 0.240 | | 0.158 | | 0.116 | 0.022 | | |
| *Overall d* |  | |  | |  | 0.015 | | |
|  |  | |  | |  |  | | |
| **History of CVDS** |  | |  | |  |  | | |
| NoxLifestyle0 | 2.73±2.67 | | 4.06±3.33 | | 3.56±3.29 | 5.98±3.41 | | |
| NoxLifestyle1 | 2.67±2.47 | | 3.64±2.79 | | 3.73±2.92 | 5.57±3.12 | | |
| NoxLifestyle2 | 3.06±2.48 | | 3.34±2.68 | | 4.20±3.05 | 5.04±3.26 | | |
| YesxLifestyle0 | 2.24±2.66 | | 1.42±2.88 | | 2.86±3.32 | 2.92±3.00 | | |
| YesxLifestyle1 | 3.56±2.87 | | 3.11±2.63 | | 4.59±2.93 | 5.17±3.03 | | |
| YesxLifestyle2 | 4.00±2.40 | | 3.14±3.01 | | 5.24±2.61 | 4.93±3.13 | | |
|  |  | |  | |  |  | | |
| *p* |  | | LS0xNo vs LS0xYes=0.001, d=0.021 | |  | LS0xNo vs LS0xYes<0.001,  d=0.021 | | |
| *Overall p* | 0.115 | | 0.040 | | 0.132 | 0.037 | | |
| *Overall d* |  | | 0.013 | |  | 0.013 | | |
|  |  | |  | |  |  | | |
| **Alcohol consumption** |  | |  | |  |  | | |
| NoxLifestyle0 | 2.83±2.77 | | 3.92±3.34 | | 3.71±3.37 | 5.64±3.55 | | |
| NoxLifestyle1 | 2.80±2.54 | | 3.38±2.66 | | 3.94±3.00 | 5.33±3.00 | | |
| NoxLifestyle2 | 3.34±2.44 | | 2.96±2.53 | | 4.49±3.03 | 4.40±3.06 | | |
| CurrentxLifestyle0 | 2.23±2.17 | | 2.47±3.29 | | 2.79±2.80 | 4.53±3.14 | | |
| CurrentxLifestyle1 | 2.40±2.31 | | 4.02±2.95 | | 3.23±2.61 | 5.84±3.40 | | |
| CurrentxLifestyle2 | 2.56±2.42 | | 4.07±2.80 | | 3.71±3.01 | 6.16±3.26 | | |
| FormerxLifestyle0 | 2.21±2.12 | | 4.00±4.05 | | 3.57±3.11 | 6.50±4.18 | | |
| FormerxLifestyle1 | 3.24±2.44 | | 4.07±3.31 | | 4.41±3.39 | 6.40±3.18 | | |
| FormerxLifestyle2 | 4.05±2.89 | | 1.60±3.05 | | 4.95±3.12 | 4.20±3.77 | | |
|  |  | |  | |  |  | | |
| *p* | LS2xNo vs LS2xCurrent=0.001, d=0.007; LS2xCurrent vs LS2xFormer=0.034, d=0.007 | |  | | LS0xNo vs LS0xCurrent=0.014, d=0.004; LS1xNo vs LS1xCurrent=0.003, d=0.005; LS2xNo vs LS2xCurrent=0.011, d=0.004 | LS2xNo vs LS2xCurren  t=0.009, d=0.018 | | |
| *Overall p* | 0.361 | | 0.054 | | 0.959 | 0.181 | | |
|  |  | |  | |  |  | | |
| **Family history of heart diseases/ stroke** |  | |  | |  |  | | |
| NonexLifestyle0 | 1.99±2.04 | | 0.91±2.21 | | 2.24±2.42 | 1.91±3.18 | | |
| NonexLifestyle1 | 1.84±2.25 | | 2.13±3.12 | | 2.40±2.70 | 3.39±4.00 | | |
| NonexLifestyle2 | 2.54±2.62 | | 2.80±3.49 | | 3.22±3.26 | 4.27±4.43 | | |
| BothxLifestyle0 | 3.36±2.95 | | 5.58±3.18 | | 4.65±3.55 | 7.23±3.03 | | |
| BothxLifestyle1 | 3.38±2.53 | | 4.91±2.77 | | 4.78±2.93 | 6.75±2.91 | | |
| BothxLifestyle2 | 3.28±2.41 | | 4.13±2.92 | | 4.78±2.94 | 6.01±3.45 | | |
| HeartxLifestyle0 | 2.68±2.88 | | 2.23±2.81 | | 3.88±3.44 | 4.59±2.63 | | |
| HeartxLifestyle1 | 2.65±2.31 | | 2.62±2.42 | | 3.85±2.55 | 4.64±2.76 | | |
| HeartxLifestyle2 | 2.92±2.24 | | 2.22±1.81 | | 4.04±2.72 | 3.68±2.20 | | |
| StrokexLifestyle0 | 3.79±2.81 | | 3.70±2.95 | | 4.83±3.46 | 6.00±3.74 | | |
| StrokexLifestyle1 | 3.60±2.67 | | 4.46±2.22 | | 4.85±2.95 | 6.68±2.49 | | |
| StrokexLifestyle2 | 4.25±2.56 | | 4.23±2.54 | | 5.67±2.94 | 6.19±3.01 | | |
|  |  | |  | |  |  | | |
| *p* | LS0xNone vs LS0xBoth<0.001, d=0.020; LS0xNone vs LS0xStroke<0.001, d=0.020; LS0xHeart vs LS0xStroke=0.011, d=0.020; LS1xNone vs LS1xBoth<0.001, d=0.034; LS1xNone vs Heart<0.001, d=0.034; LS1xNone vs LS1xStroke<0.001, d=0.034; LS1xBoth vs LS1xHeart=0.002, d=0.034; LS1xHeart vs LS1xStroke=0.002, d=0.034; LS2xNone vs LS2xStroke<0.001, d=0.012; LS2xBoth vs LS2xStroke=0.033, d=0.012; LS2xHeart vs LS2xStroke<0.001, d=0.012 | |  | | LS0xNo vs LS0xBoth<0.001, d=0.038; LS0xNo vs LS0xHeart<0.001, d=0.038; LS0xNo vs LS0xStroke<0.001, d=0.038; LS1xNo vs LS1xBoth<0.001, d=0.053; LS1xNo vs LS1xHeart<0.001, d=0.053; LS1xNo vs LS1xStroke<0.001, d=0.053; LS1xBoth vs LS1xHeart<0.001, d=0.053; LS1xHeart vs LS1xStroke=0.008, d=0.053; LS2xNo vs LS2xBoth<0.001, d=0.019; LS2xNo vs LS2xStroke<0.001, d=0.019; LSxHeart vs LS2xStroke<0.001, d=0.019 | LS0xNo vs LS0xBoth<0.001,  d=0.073;  LS0xNo vs LS0xHeart<0.001,  d=0.073; LS0xNo vs LS0xStroke  =0.010,  d=0.073; LS1xNo vs LS1xBoth  <0.001,  d=0.085; LS1xNo vs LS1xHeart  =0.001,  d=0.085; LS1xNo vs LS1xStroke  <0.001,  d=0.085; LS1xBoth vs Heart  =0.001,  d=0.085; LS1xHeart vs LS1  xStroke=0.005,  d=0.085; LS2xNo vs  LS2xBoth  =0.004,  d=0.045; LS2xNo vs  LS2xStroke  =0.003,  d=0.045; LS2xBoth vs  LS2xHeart  =0.006,  d=0.045; LS2xHeart vs LS2xStroke  =0.007,  d=0.045 | | |
| *Overall p* | 0.418 | | 0.219 | | 0.320 | 0.162 | | |
|  |  | |  | |  |  | | |
| **History of HIV/AIDS** |  | |  | |  |  | | |
| NoxLifestyle0 | 2.69±2.69 | | 3.53±3.39 | | 3.46±3.31 | 5.33±3.38 | | |
| NoxLifestyle1 | 2.66±2.54 | | 2.75±2.85 | | 3.61±2.93 | 4.52±3.31 | | |
| NoxLifestyle2 | 3.05±2.51 | | 2.30±2.25 | | 4.04±3.02 | 3.66±2.66 | | |
| YesxLifestyle0 | 2.98±2.44 | | 3.95±3.46 | | 4.43±2.83 | 5.95±3.95 | | |
| YesxLifestyle1 | 2.93±2.13 | | 4.39±2.44 | | 4.62v2.73 | 6.50±2.54 | | |
| YesxLifestyle2 | 3.29±2.28 | | 5.06±2.51 | | 5.43±2.98 | 7.36±2.81 | | |
|  |  | |  | |  |  | | |
| *p* |  | | LS1xNo vs LS1xYes<0.001, d=0.053; LS2xNo vs LS2xYes<0.001, d=0.065 | | LS0xNo vs LS0xYes=0.042, d=0.002; LS1xNo vs LS1xYes<0.001, d=0.007; LS2xNo vs LS2xYes<0.001, d=0.006 | LS1xNo vs LS1xYes  <0.001,  d=0.057;  LS2xNo vs LS2xYes  <0.001, d=0.089 | | |
| *Overall p* | 0.993 | | 0.046 | | 0.681 | 0.006 | | |
| *Overall d* |  | | 0.012 | |  | 0.020 | | |
|  |  | |  | |  |  | | |
| **History of psychiatric diseases** |  | |  | |  |  | | |
| NoxLifestyle0 | 2.71±2.67 | | 3.64±3.39 | | 3.53±3.29 | 5.49±3.52 | | |
| NoxLifestyle1 | 2.70±2.49 | | 3.75±2.80 | | 3.76±2.93 | 5.78±3.10 | | |
| NoxLifestyle2 | 3.09±2.48 | | 4.23±2.95 | | 4.23±3.04 | 6.43±3.26 | | |
| YesxLifestyle0 | NA | | NA | | NA | NA | | |
| YesxLifestyle1 | NA | | 1.45±0.95 | | NA | 2.45±0.51 | | |
| YesxLifestyle2 | NA | | 1.73±0.87 | | NA | 2.56±0.90 | | |
|  |  | |  | |  |  | | |
| *p* |  | | LS1xNo vs LS1xYes=0.007, d=0.014; LS2xNo vs LS2xYes<0.001, d=0.026 | |  | LS1xNo vs LS1xYes<0.001,  d=0.023;  LS2xNo vs LS2xYes<0.001,  d=0.074 | | |
| *Overall p* | NA | | 0.773 | | NA | 0.584 | | |
|  |  | |  | |  |  | | |
| **CALCULATED RISK FACTORS** |  | |  | |  |  | | |
| **BMI** |  | |  | |  |  | | |
| UnderweightxLifestyle0 | 3.15±2.74 | | NA | | 4.60±3.69 | NA | | |
| UnderweightxLifestyle1 | 2.73±2.74 | | 5.00±3.27 | | 3.73±3.53 | 6.86±3.76 | | |
| UnderweightxLifestyle2 | 2.35±2.21 | | 2.80±3.35 | | 3.17±2.74 | 3.60±3.51 | | |
| NormalxLifestyle0 | 2.83±2.84 | | 4.58±3.25 | | 3.68±3.38 | 6.44±3.28 | | |
| NormalxLifestyle1 | 2.95±2.65 | | 3.95±2.68 | | 3.97±3.01 | 5.71±3.09 | | |
| NormalxLifestyle2 | 3.04±2.51 | | 3.41±2.69 | | 4.20±3.02 | 5.10±3.21 | | |
| OverweightxLifestyle0 | 2.79±2.61 | | 3.18±3.50 | | 3.63±3.37 | 4.82±3.49 | | |
| OverweightxLifestyle1 | 2.66±2.30 | | 3.58±2.70 | | 3.75±2.78 | 5.72±2.90 | | |
| OverweightxLifestyle2 | 3.37±2.45 | | 3.09±2.71 | | 4.60±3.07 | 5.09±3.42 | | |
| ObesexLifestyle0 | 2.33±2.34 | | 2.38±3.20 | | 3.00±2.89 | 4.38±3.64 | | |
| ObesexLifestyle1 | 2.14±2.21 | | 2.35±2.71 | | 3.24±2.72 | 4.53±3.20 | | |
| ObesexLifestyle2 | 2.86±2.41 | | 3.24±2.74 | | 3.86±3.11 | 4.81±3.31 | | |
|  |  | |  | |  |  | | |
| *p* | LS1xNormal vs LS1xObese=0.001, d=0.006 | | LS0xNormal vs LS0xObese=0.006, d=0.021; LS1xNormal vs LS1xObese<0.001, d=0.032 | | LS1xNormal vs LS1xObese=0.034, d=0.004 | LS0xNormal vs LS0xObese  =0.007, d=0.020;  LS1xNormal vs  LS1xObese<0.001,  d=0.033;  LS1xOverweight vs LS1xObese  =0.040, d=0.033 | | |
| *Overall p* | 0.269 | | 0.136 | | 0.231 | 0.139 | | |
|  |  | |  | |  |  | | |

MI: myocardial infarction, NA: not applicable, CVDS: cardiovascular diseases (dyslipidemia, heart diseases, stroke or diabetes), *d*: effect size, psychiatric diseases: anxiety or depression, Lifestyle behaviours: include any of the following i.e., no smoking, healthy diet or physical activity (each count as 1 lifestyle behaviour),

LS0: no lifestyle behaviour, LS1: one lifestyle behaviour, LS2: 2 or more lifestyle behaviours, , BMI: body mass index, 1: age 18-34 years, 2: 35-49 years, 3:>50 years
