# Supplementary material for: Age and healthy lifestyle behavior’s disparities and similarities on knowledge of myocardial infarction symptoms and risk factors among public and outpatients in a resource-limited setting, cross-sectional study in greater Gaborone, Botswana
Source: BMC Cardiovasc Disord. 2024 Mar 4;24:140. doi: 10.1186/s12872-024-03792-4 (PMC10910839; doi:10.1186/s12872-024-03792-4)

eFigure 3a. Public myocardial infarction symptoms knowledge scores stratified by number of healthy lifestyle behaviors

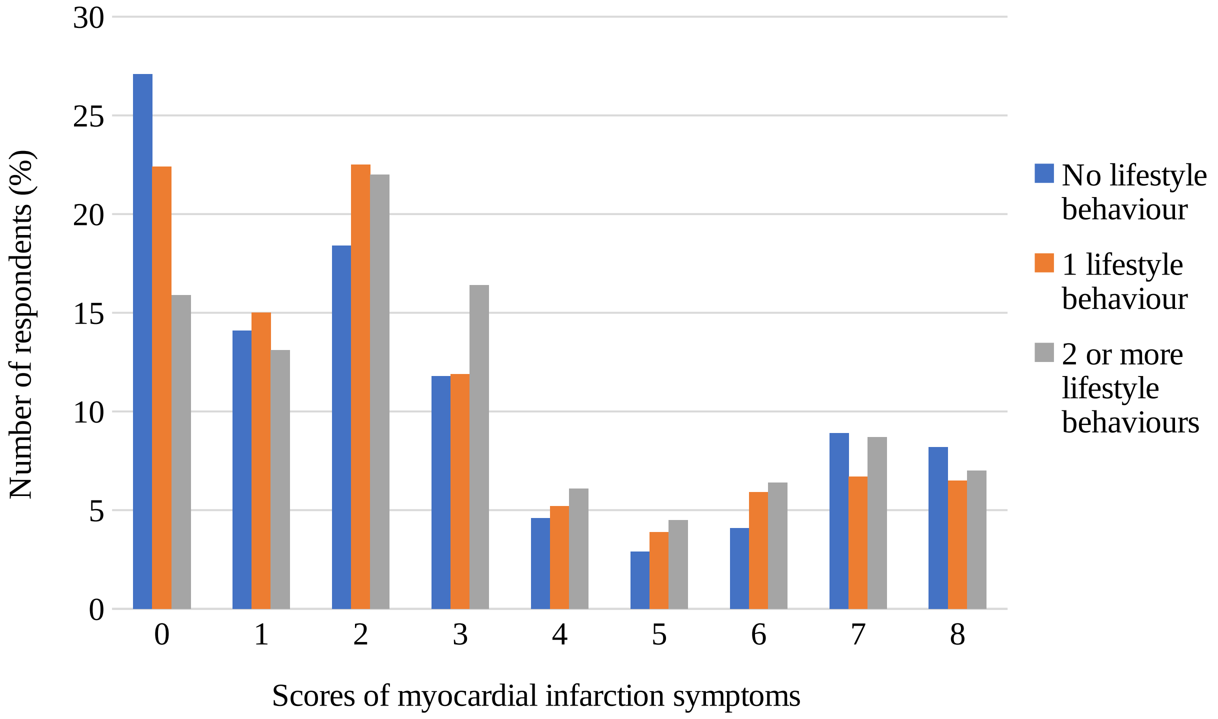

eFigure 3b. Outpatients myocardial infarction symptoms knowledge scores stratified by number of healthy lifestyle behaviors

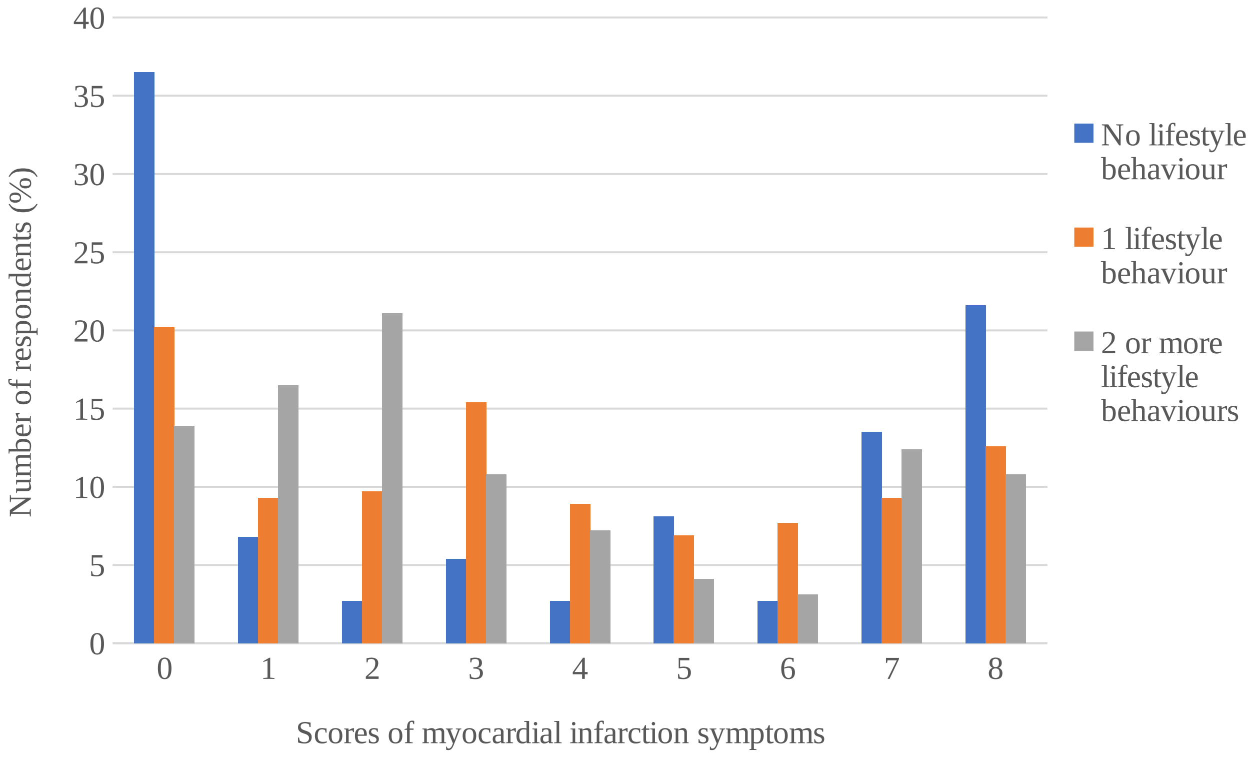

Supplement: Supplementary file 7 [file 12872_2024_3792_MOESM3_ESM.docx]
