# Supplementary material for: Age and healthy lifestyle behavior’s disparities and similarities on knowledge of myocardial infarction symptoms and risk factors among public and outpatients in a resource-limited setting, cross-sectional study in greater Gaborone, Botswana
Source: BMC Cardiovasc Disord. 2024 Mar 4;24:140. doi: 10.1186/s12872-024-03792-4 (PMC10910839; doi:10.1186/s12872-024-03792-4)

eFigure 4a. Public myocardial infarction risk factors knowledge stratified by number of lifestyle behaviours

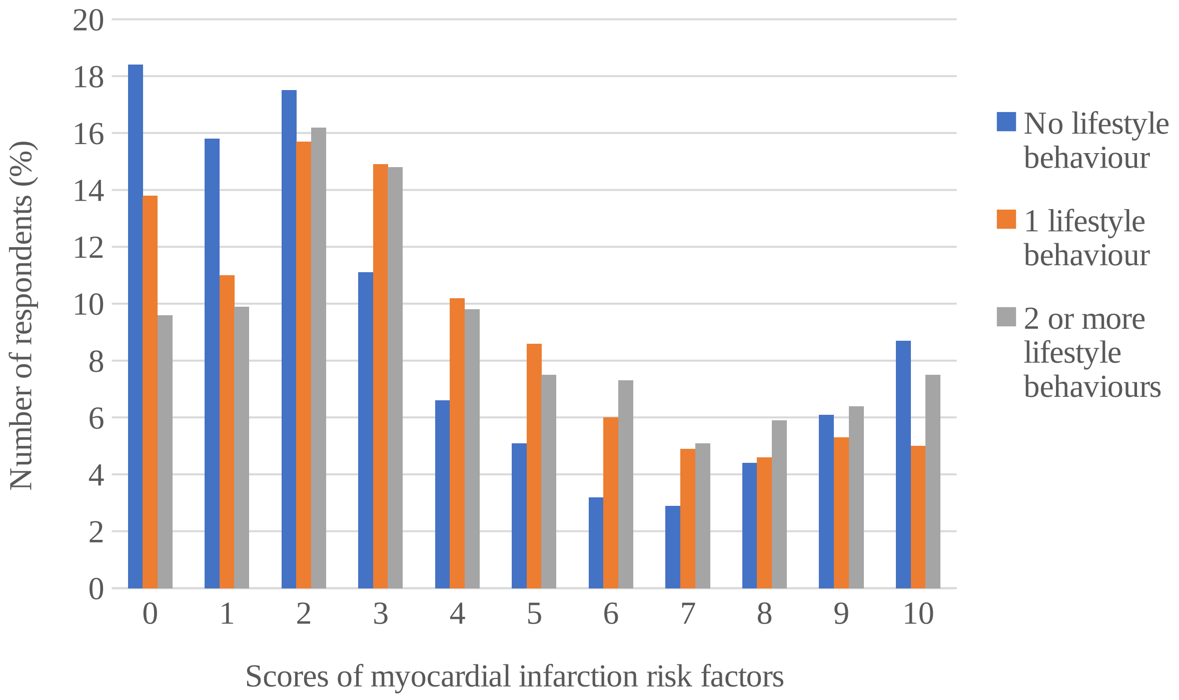

eFigure 4b. Outpatients myocardial infarction risk factors knowledge scores stratified by number of lifestyle behaviours

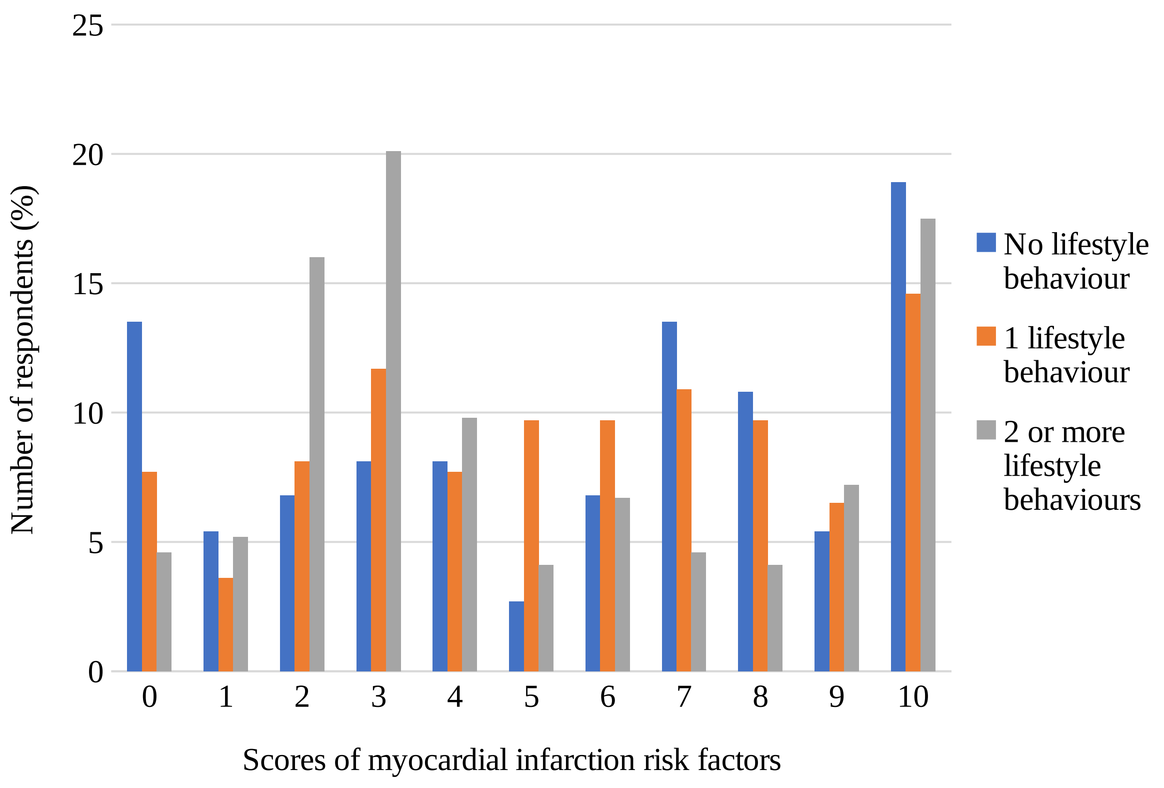

Supplement: Supplementary file 8 [file 12872_2024_3792_MOESM4_ESM.docx]
